# Protein cleaver: an interactive web interface for *in silico* prediction and systematic annotation of protein digestion-derived peptides

**DOI:** 10.3389/fbinf.2025.1576317

**Published:** 2025-09-04

**Authors:** Grigorios Koulouras, Yingrong Xu

**Affiliations:** 1 Pfizer Center for Digital Innovation, Thessaloniki, Greece; 2 Pfizer Worldwide Research and Development, Groton, CT, United States

**Keywords:** proteolytic digestion, peptide annotation, peptide identification, mass spectrometry, proteomics

## Abstract

Proteolytic digestion is an essential process in mass spectrometry-based proteomics for converting proteins into peptides, hence crucial for protein identification and quantification. In a typical proteomics experiment, digestion reagents are selected without prior evaluation of their optimality for detecting proteins or peptides of interest, partly due to the lack of comprehensive and user-friendly predictive tools. In this work, we introduce Protein Cleaver, a web-based application that systematically assesses regions of proteins that are likely or unlikely to be identified, along with extensive sequence and structure annotation and visualization features. We showcase practical examples of Protein Cleaver’s usability in drug discovery and highlight proteins that are typically difficult to detect using the most common proteolytic enzymes. We evaluate trypsin and chymotrypsin for identifying G-protein-coupled receptors and discover that chymotrypsin produces significantly more identifiable peptides than trypsin. We perform a bulk digestion analysis and assess 36 proteolytic enzymes for their ability to detect most of cysteine-containing peptides in the human proteome. We anticipate Protein Cleaver to be a valuable auxiliary tool for proteomics scientists.

## Introduction

Proteolytic digestion is a cornerstone in mass spectrometry-based proteomics experiments as it allows for the systematic breakdown of proteins into smaller, more manageable peptides. This process is essential for the identification and quantification of proteins in complex biological samples. Proteolytic enzymes, known as proteases, cleave proteins at specific sites, generating peptides that are unique to the parent protein. These peptides can then be analyzed by mass spectrometry, which measures their mass-to-charge ratio to determine their identity and abundance. This method is highly sensitive and can detect thousands of peptides in a single run, making it invaluable for proteomics research where the goal is to catalog and understand protein expression, function, and structure. Furthermore, proteolytic digestion is crucial for studying post-translational modifications and protein-protein interactions, providing insights into the dynamic nature of the proteome and its role in various diseases and biological processes. Although there are many proteases with well-established cleavage rules, the scientific community often relies on only a few of them in their experiments. Trypsin is the gold-standard protease for mass spectrometry (MS)-based proteomics due to its low price, widespread availability and the ability to cleave at the carboxyl terminal of arginine and lysine residues, except when either is followed by proline, resulting in a positively charged peptide C-terminus (Huang et al., 2005; Dongré et al., 1996). Trypsin’s specificity is high, and it produces peptides with an average atomic mass of 700–1500 Da which is the ideal range for mass spectrometry analysis. However, other proteases are often utilized to obtain additional data (Swaney et al., 2010; Giansanti et al., 2016). Among these, the endoproteinases AspN and GluC target acidic amino acid residues and generate peptide mixtures of similar complexity to those produced by trypsin and have been successfully used in other studies (Dau et al., 2020; Fossati et al., 2021; Swaney et al., 2010). Chymotrypsin, which primarily targets aromatic residues, has also been utilized (Giansanti et al., 2016). In contrast, broad specificity proteases are less commonly used in proteomics due to the high complexity of the peptide mixtures they generate. Experimental validation of all available proteolytic enzymes in a given experiment is costly, time consuming and practically impossible, making trypsin and few other proteases the enzymes of choice.

To this end, several software tools have been developed to predict cleavage sites of proteases in protein sequences. Notably, PeptideCutter (Wilkins et al., 1999) from the Expasy bioinformatics resource portal and the Andromeda peptide search engine integrated into MaxQuant (Cox et al., 2011; Cox and Mann, 2008) are among the most widely used. Recently, significant development efforts have resulted in the development of more sophisticated tools with expanded functionalities. The Rapid Peptides Generator (Maillet, 2019) allows users to simultaneously select multiple proteases and create custom cleavage rules. ProsperousPlus (Li et al., 2023) is pre-loaded with models for 110 protease types and among with iProt-Sub (Song et al., 2019) and Prosperous (Song et al., 2018) have been employed in several studies. Procleave (Li et al., 2020a) and Deepcleave (Li et al., 2020b) are two additional bioinformatics methods for predicting caspase and matrix metalloprotease substrates and cleavage sites. The latter is the first deep learning approach for substrates and cleavage sites prediction based on a predictive model that employs convolutional neural networks with transfer learning. A detailed list of existing methods for predicting protease-specific substrates and cleavage sites is available (Li et al., 2019), and an in-depth review of statistical methods for predicting proteolytic cleavage has been previously published (duVerle and Mamitsuka, 2012).

Nonetheless, there is room for improvement as no existing platform currently integrates protease-derived peptide predictions with comprehensive amino acid-level peptide annotation and sequence-to-structure mapping. In this paper, we present Protein Cleaver, an interactive web-based application developed using the Shiny package, which can be executed on any laptop or personal computer that supports R. It is platform independent and available as open-source software, allowing it to be deployed on any Shiny Server and accessed through a browser. Protein Cleaver builds upon the cleaver R package (Gibb, 2024), available in Bioconductor, which offers a fast and accurate framework for cleavage rules and exceptions for 36 proteolytic enzymes, as described on the Expasy web server (https://web.expasy.org/peptide_cutter/) (Wilkins et al., 1999). Protein Cleaver performs *in silico* protein digestion and provides users with a list of identifiable peptides for a given set of proteins. It visualizes regions of proteins that are more or less likely to be identified in both primary and tertiary structures. Protein Cleaver’s user interface combines the neXtProt sequence viewer (Zahn-Zabal et al., 2020) and the MolArt structural viewer (Hoksza et al., 2018), offering a comprehensive platform for evaluating peptides of interest. It also encompasses various co-occurring elements, including disulfide bonds, known post-translational modifications, secondary structure elements, and disease-associated variants, all retrieved in real-time from UniProt. This mechanism ensures automatic synchronization of annotations with the latest UniProt data. Additionally, it provides structural annotations derived from the Protein Data Bank, the AlphaFold Protein Structure DB, or the SWISS-MODEL Repository when no experimentally determined three-dimensional (3D) structures are available. Protein Cleaver provides a bulk digestion feature that systematically assesses all available proteases to determine their optimality for a set of uploaded proteins. This key aspect of Protein Cleaver enables users to conduct an *in silico* proteome-wide analysis in advance and then choose the most suitable proteases for their experiment. We evaluate Protein Cleaver’s usability in G-protein-coupled receptors (Rosenbaum et al., 2009), the largest and most diverse group of membrane receptors in eukaryotes which are promising drug targets. Our findings demonstrate that chymotrypsin (high specificity) is more effective than trypsin in identifying a greater number of peptides for this specific protein group. According to the cleavage rules and exceptions table provided in the Expasy web page (https://web.expasy.org/peptide_cutter/peptidecutter_enzymes.html), chymotrypsin (high specificity) preferentially cleaves at aromatic residues tryptophan, tyrosine, and phenylalanine at the P1 position, and to a lesser extent at leucine, methionine, and histidine under low-specificity conditions. While high-specificity cleavage is valuable for theoretical predictions, it can be difficult to replicate experimentally. To address this, Protein Cleaver offers both high- and low-specificity chymotrypsin options, with the latter providing a more realistic simulation of *in vitro* digestion patterns. We showcase that peptides identifiable with chymotrypsin (high specificity) are predominantly located in transmembrane domains. Due to the lack of charged amino acid residues like lysine or arginine, transmembrane domains are theoretically less accessible and challenging to detect using trypsin. Furthermore, we systematically assess five of the most frequently used proteases and investigate the detectability of cysteine-carrying peptides. This is essential for studies focusing on post-translational modifications, such as cysteine-oxidation, aiming to uncover the maximum number of peptides that contain specific amino acids. In addition, we perform a bulk digestion using all available proteases and assess the entire proteomes of *Homo sapiens* and *Saccharomyces cerevisiae*. Our findings indicate that neutrophil elastase has the potential to reveal more peptides and cover a larger portion of the human proteome (42,466 out of the total 42,517 reviewed proteins including isoforms) compared to trypsin (42,403 identifiable proteins), which ranks as the third most effective proteolytic enzyme (Supplementary Figures S1–S4). However, it is worth noting that neutrophil elastase is a broad specificity protease which cleaves proteins at multiple amino acid sites. This may lead to more peptides and theoretically higher proteome coverage, but these peptides might be less unique to the parent proteins, therefore less informative. Lastly, we demonstrate that a few proteins in the human and yeast proteomes are likely undetectable using trypsin, either due to the absence of cleavable sites or because the resulting peptides fall outside the optimal length range for detection.

## Materials and methods

Protein Cleaver is a web-based application written in Shiny, compatible with any operating system that supports R. The application accepts a multi-fasta file or a list of UniProt Accessions as input, along with parameters that define the minimum and maximum length and mass of peptides to be considered upon digestion. Users can also specify the number of allowed miscleavages to simulate real-world experimental conditions and choose from 36 available proteases. Miscleavages are a crucial aspect of proteomics research, where proteins may not be cleaved at the expected sites due to biological, chemical, or other undefined conditions (Chiva et al., 2014). Protein Cleaver lists peptides that are likely or unlikely to be identified. Identifiable peptides are categorized as either unique to their parent protein or shared across multiple proteins in the uploaded set. This segregation is useful for research questions involving peptides that are not common between proteins. Peptide summary statistics are provided in a separate tab, presenting the frequency of each peptide and the number of proteins it belongs to.

**FIGURE 1:**
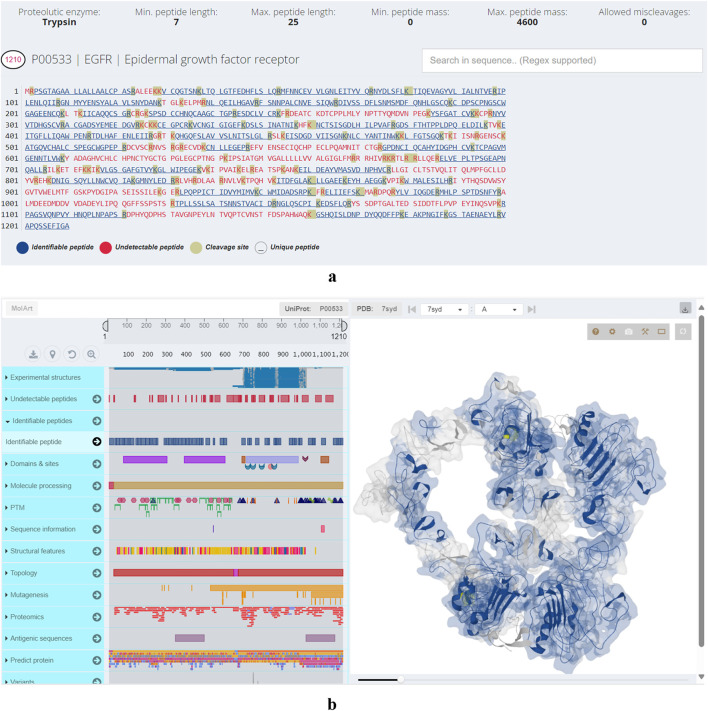
**(a)** Visual representation of epidermal growth factor receptor primary structure. Motifs in blue denote identifiable peptides, motifs in red undetectable while the amino acids amino acids highlighted in grey color are cleavage sites. **(b)** Structural viewer in Protein Cleaver. Left-hand panel: hover over primary structure of epidermal growth factor receptor to identify co-occurring elements (post-translational modifications, structural features, domains, sites, variants) from UniProt in real-time. Right-hand panel: visualizes user selections in the 3D structure.

Additionally, the user interface lists identifiable proteins and proteins hard to detect, along with calculated data such as the total number of peptides from an ideal proteolytic digestion, the predicted number of identifiable peptides, and the sequence coverage if all identifiable peptides are detected. Users can visually inspect protein sequences for regions that are likely to be undetected. An integrated sequence viewer highlights detectable peptides, those unlikely to be observed, and the protein’s cleavage sites (Figure 1a). Similarly, a structural viewer has been incorporated to provide users with a comprehensive view of the resulting peptides (Figure 1b). Both viewers are interconnected, allowing users to explore regions in the sequence panel while the corresponding area in the structure is highlighted and *vice versa*.

Protein Cleaver has been designed with interpretability as a key focus. As a result, it features a summary statistics section where users can swiftly examine the frequency of cleaved peptides by peptide length, the contribution of fully cleaved peptides to the total sequence coverage of the uploaded protein set by peptide length, and the frequency of detectable amino acids across the entire dataset. This feature is particularly useful for users interested in specific amino acids, such as when studying post-translational modifications.

Additionally, the bulk digestion feature allows for a comprehensive assessment of all available proteases against the uploaded protein set. It ranks proteases based on the overall maximum coverage achievable (considering all detectable regions of all proteins over the length of all uploaded protein sequences) and the number of identifiable peptides, providing insights into the optimal enzyme for the uploaded protein sequences. However, this estimation assumes maximum coverage and peptide count without considering whether these peptides are unique or shared across multiple proteins, so it should be used with caution.

## Results

### Trypsin vs. chymotrypsin for G-protein-coupled receptors

**FIGURE 2:**
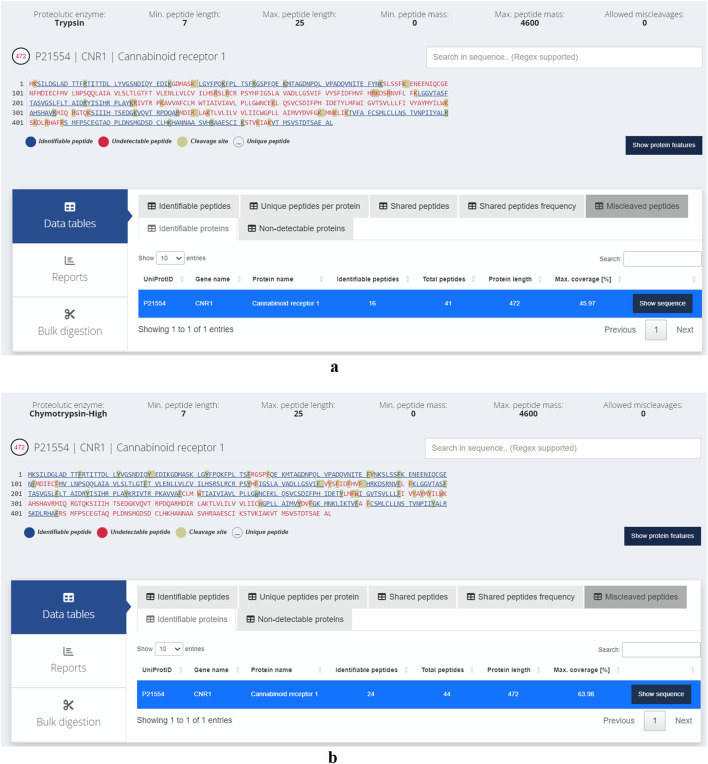
**(a)** Identifiable (in blue) and hard to detect (in red) peptides for CNR1 using trypsin. The cleavage sites are highlighted in grey. No miscleavages have been considered, assuming an ideal proteolytic digestion. Approximately 46% of the protein sequence can be theoretically detected. **(b)** Identifiable (in blue) and hard to detect (in red) peptides for CNR1 using chymotrypsin (high specificity). The cleavage sites are highlighted in grey. No miscleavages have been considered, assuming an ideal proteolytic digestion. Approximately 64% of the protein sequence can be theoretically identified.

**FIGURE 3:**
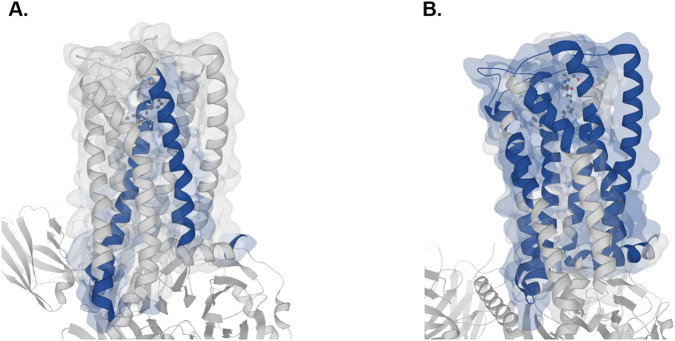
3D visual representation of the identifiable peptides (highlighted in blue) for CNR1 with trypsin **(A)** and chymotrypsin **(B)** The 8GHV structure from PDB has been selected. The prediction was made by Protein Cleaver and visualized in MolArt, which is integrated into it.

GPCRs (G-protein-coupled receptors) are highly attractive drug targets and 33% of small-molecule drugs target GPCRs (Santos et al., 2017). Elucidating the structures and functions of GPCRs would significantly accelerate drug discovery. Mass spectrometry (MS) has emerged as a powerful tool for GPCR characterization due to its low sample requirements and relatively fast analysis time. In addition to the protein sequences, MS-based approaches can provide insights into post-translational modifications, ligand binding, protein interactors, and signaling pathways (Alfonzo-Mendez et al., 2016; Yen et al., 2023). GPCRs, like other membrane proteins, are challenging to study with bottom-up proteomics. This is due to their low abundance, high hydrophobicity and lack of charged residues such as lysine and arginine, which are cleaved by trypsin or endoproteinase Lys-C, the most widely used proteases in bottom-up proteomics (Barrera and Robinson, 2011). The transmembrane (TM) domains of GPCRs are abundant in hydrophobic residues and rarely contain lysine and arginine, thus hindering digestion by trypsin. Alternative proteases can be used to improve coverage of the TM domains. For example, chymotrypsin cleaves peptide bonds at the carboxyl side of aromatic amino acid residues such as tryptophan, phenylalanine and tyrosine (Ingles and Knowles, 1967). In a previous study, chymotrypsin was used to improve sequence coverage of GPCRs including human cannabinoid receptor 1 (CNR1) (Zvonok et al., 2010). In lieu of experimentally testing the proteases, *in silico* digestion offers a compelling alternative to predict the suitability of proteases. Here, we assess the ability of trypsin and chymotrypsin (high specificity) in cleaving CNR1 and demonstrate the ease of using Protein Cleaver for this purpose. CNR1 (UniProt ID: P21554) was selected which contains 472 amino acids. Of these, 44 are either lysine or arginine, making up approximately 9.2% of the sequence. While trypsin is expected to ideally cleave CNR1, this is only partially true. Chymotrypsin (high specificity) is an alternative enzyme that can potentially cleave CNR1 into more theoretically identifiable peptides, thereby offering broader protein coverage (Figures 2a,b). Assuming an ideal digestion, without any miscleavages, approximately 46% of the protein sequence can be theoretically detected using trypsin. In contrast, chymotrypsin seems to cover 64% of the sequence, making it more suitable for detecting the specific protein. Another aspect that can be easily inferred using the integrated 3D structure viewer is the localization of the peptides predicted to be detected. Chymotrypsin can detect eight additional peptides compared to trypsin, and interestingly, most of those belong to TM domains, therefore hard to be detected using trypsin (Figure 3). Additional analysis with similar findings has been conducted for the Cannabinoid receptor (CNR1, UniProt ID: P21554), the Mu-type opioid receptor (OPRM1, UniProt ID: P35372) and the Neurotensin receptor type 1 (NTSR1, UniProt ID: P30989). The results are presented in (Supplementary Figures S5–S10). These examples illustrate the usability of Protein Cleaver for research questions that require a detailed and focused examination of a specific protein or its regions.

### Amino acids detectability in whole proteomes

**FIGURE 4:**
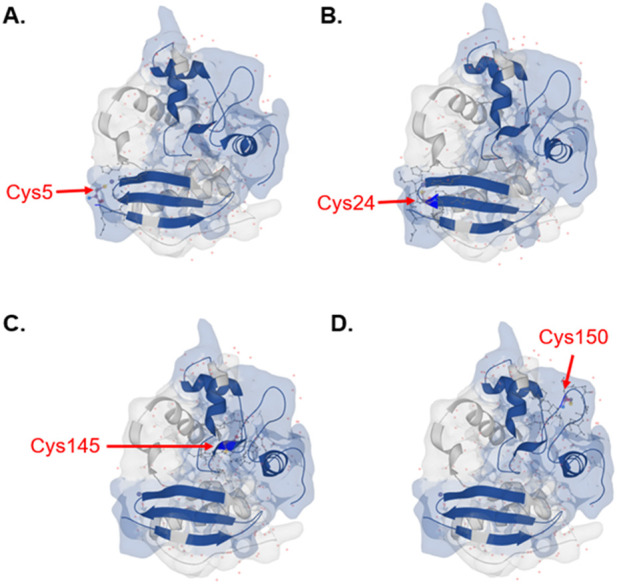
Detectable cysteines with trypsin predicted by Protein Cleaver for MGMT highlighted in the integrated 3D structure viewer. Cysteine positions are **(A)** Cys5, **(B)** Cys24, **(C)** Cys145 and **(D)** Cys150. Detectable peptides were shown in blue color. The 1EH6 structure from PDB has been selected.

In addition to *in silico* digestion of individual proteins, Protein Cleaver can also be utilized to examine digestion profiles of entire proteomes using a protease of choice. Among the 20 protein-encoding amino acids, cysteine stands out due to its high nucleophilicity and redox sensitivity (Maurais and Weerapana, 2019; van der Reest et al., 2018). Cysteine is especially attractive for drug discovery owing to its key role in regulating protein function, it is highly conserved at functional sites of a broad range of enzymes despite its low abundance (Maurais and Weerapana, 2019; van der Reest et al., 2018). Cysteine is the main target of fragment-based drug discovery and reactive cysteine profiling has played a key role in covalent drug discovery (van der Reest et al., 2018; Bonifácio et al., 2021; Weerapana et al., 2010; Murray and Rees, 2009). Protein Cleaver can be used to calculate proteome-wide amino acid detectability and visualize detectable residues in protein structures, facilitating studies of cysteine by MS-based proteomics. Calculated by Protein Cleaver, 56.98% of cysteine-containing peptides generated by trypsin are detectable by MS, as compared to 49.77% by chymotrypsin and 41.74% by Glu-C (Supplementary Figures S11–S15). Besides global amino acid detectability analysis, Protein Cleaver can also provide structural details of detectable residues. For instance, all four detectable cysteines, including the active site Cys145 from MGMT (methylated-DNA--protein-cysteine methyltransferase), were predicted by Protein Cleaver with trypsin as the enzyme of choice and can be visualized in the protein’s 3D structure (Figure 4). Understanding the MS-detectable cysteines and their locations in protein structures would facilitate reactive cysteine research and covalent drug discovery.

### Proteins with low detectability across entire proteomes

**FIGURE 5:**
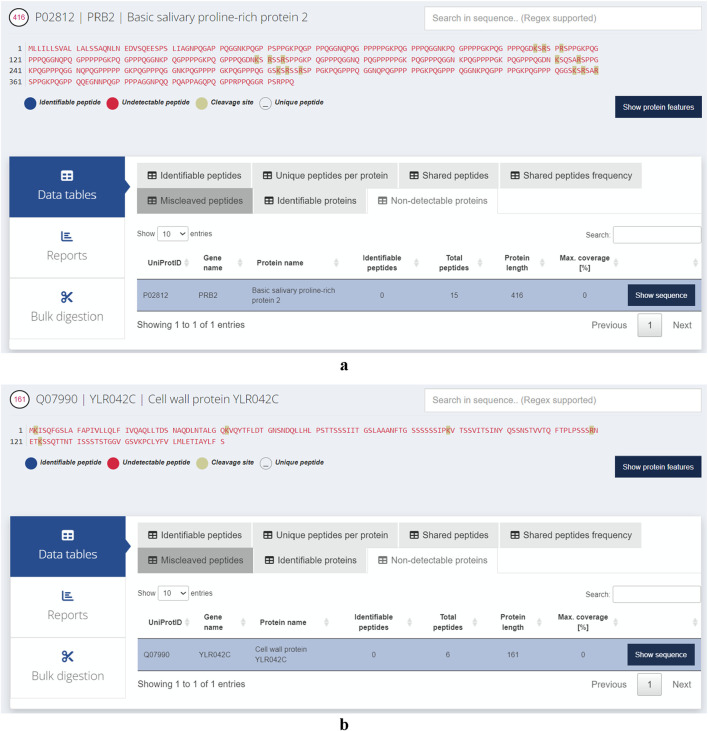
**(a)** The longest protein likely to remain undetected in *Homo sapiens* is PRB2, as its digested peptides tend to fall outside the optimal length range for detection in mass spectrometry-based experiments. **(b)** The longest protein likely to remain undetected in *Saccharomyces cerevisiae* is cleaved by trypsin into six peptides that are either too short for unique mapping or too long for efficient detection by standard proteomics.

We assessed the likelihood of proteins being undetectable using trypsin and the most common parameters used in various bottom-up proteomics software. These programs require users to define the minimum and maximum peptide lengths, as well as the minimum and maximum peptide masses. Typically, the minimum length is set at seven amino acids and the maximum at 25, while the mass ranges from 0 to 4,600 Da. Interestingly, our analysis revealed that 114 proteins (out of the 42,517 reviewed records including isoforms) in the *H. sapiens* proteome are likely to remain undetected with these default settings (Supplementary Table S1). For *S. cerevisiae*, 19 proteins are undetectable using the same parameters out of the total 6,091 records examined. The longest non-identified human protein is the ‘Basic salivary proline-rich protein 2'(PRB2, UniProt ID: P02812). Despite consisting of 416 amino acids, the peptides generated are either too long or too short because of their amino acid composition and the positions of arginine and lysine (Figure 5a). In *S. cerevisiae*, the longest protein that is challenging to detect is the ‘Cell wall protein’ (YLR042C, UniProt ID: Q07990). This protein is theoretically cleaved into six peptides by trypsin, but none of these peptides fall within the range of 7–25 amino acids, as illustrated in Figure 5b. To further illustrate the utility of Protein Cleaver for single-protein analysis, we performed an *in silico* tryptic digestion of Mucin-22 (MUC22, UniProt ID: E2RYF6) and compared the predicted detectable peptides with experimental MS-based data from PeptideAtlas (Protein ID: E2RYF6; Supplementary Figures S16–S17) (Desiere et al., 2006). MUC22 is predicted to be poorly detectable using trypsin, as the resulting peptides fall outside the optimal mass and length range for mass spectrometry. Notably, PeptideAtlas reports no experimentally detected peptides for MUC22, despite its considerable size (1773 amino acids), suggesting that its sequence characteristics may hinder detection in standard proteomics workflows.

### Availability of the method

Protein Cleaver is provided as open source under the GNU General Public License (GPLv3). The source code has been deposited on a public repository (https://github.com/gkoulouras/ProteinCleaver). Protein Cleaver is platform-independent and has been tested on Microsoft Edge version 131.0.2903.86 and Google Chrome version 131.0.6778.109. User documentation is available on GitHub for running Protein Cleaver locally on a personal computer. Alternatively, the source code can be deployed on a Posit server to serve Protein Cleaver as a web application.

## Discussion

In recent years, there has been a substantial rise in predictive methods and tools within the proteomics research field, including computational approaches for proteolytic digestion of proteins. In this work, we introduce Protein Cleaver, a robust rule-based tool for predicting protease-induced cleavage sites in protein sequences and assessing the performance of various proteases. The resulting peptides can effortlessly be mapped onto protein 3D structures, enabling visualization of cleaved peptides within the context of tertiary protein structures. We conducted *in silico* digestion of CNR1, a G-protein coupled receptor for endogenous cannabinoids, and demonstrated a significant improvement with chymotrypsin over trypsin in accessing transmembrane domains. While only 16 peptides were predicted to be identifiable with trypsin, a total of 24 peptides appeared to be identifiable with chymotrypsin (high specificity), highlighting the utility of Protein Cleaver for *a priori* computational evaluation of proteolytic enzymes. To validate Protein Cleaver’s predictions with real-world data, we applied it to a single-protein case study by performing an *in silico* tryptic digestion of MUC22. We then compared the predicted detectable peptides with publicly available experimental data from PeptideAtlas. Despite MUC22’s considerable length (1773 amino acids), no peptides are reported as experimentally detected in PeptideAtlas, which aligns with our prediction that the protein is poorly detectable due to suboptimal peptide properties. This comparison highlights Protein Cleaver’s practical utility and its consistency with large-scale, open-access proteomics datasets. In addition to comparing proteases for a single protein or its regions, we demonstrated how Protein Cleaver can systematically assess proteolytic digestion on a proteome-wide scale. We focused on cysteine as the amino acid of interest due to its role as a functional and regulatory hotspot, relevant to oxidative stress, chemo proteomics, and drug target discovery (Gu and Robinson, 2016). We then performed *in silico* digestion on the *H. sapiens* and *S. cerevisiae* proteomes and evaluated three available proteases in identifying the majority of cysteine-containing peptides. Trypsin appears to identify approximately 57% of the overall cysteine-containing peptides in the human proteome, while chymotrypsin and Glu-C can detect 49,77% and 41,74% respectively. Unlike other software, Protein Cleaver highlights peptides that are likely to remain undetected, listing and visualizing them within a protein’s tertiary structure. Future plans include the expansion of Protein Cleaver to allow multiple proteases for either parallel or sequential digestion with multiple proteases to simulate a multi-enzyme digestion, as well as the integration of user-defined cleavage rules to accommodate emerging proteases.

It is important to note that, beyond protease selection, factors such as sample preparation, liquid chromatography (LC) separation, and the choice of mass spectrometer also influence peptide identification. For instance, the use of detergents like sodium dodecyl sulfate or sodium deoxycholate during protein extraction can enhance the detection of membrane proteins. (Glatter et al., 2016). Longer LC gradients, or additional LC separation such as high pH reverse phase fractionation (Stein et al., 2013) can further improve peptide and protein identification. Different mass analyzers could also lead to different identifications results. The Orbitrap analyzer has high resolving power and is ideal for analyzing biomolecules such as peptides or proteins. The Time-of-flight (TOF) analyzer has high ion transmission efficiencies, therefore can achieve the widest mass range of all mass analyzers (Haag, 2016) and will have advantages over Orbitraps in analyzing larger peptides or proteins. Even with the same mass analyzer, different generations of instrumental design could impact identifications. For instance, Orbitrap Ascend Tribrid mass spectrometer could identify 76% more tryptic peptides for single-shot proteomics analysis of low input samples compared to the previous generation of the Tribrid instruments, the Orbitrap Eclipse (He et al., 2023).

While peptide mass and length are not the only factors influencing peptide identifiability, they remain foundational parameters in proteomics workflows. Widely used platforms such as MaxQuant (Cox and Mann, 2008) and Proteome Discoverer (Orsburn, 2021; https://docs.thermofisher.com/r/Proteome-Discoverer-3.2-User-Guide/) rely heavily on these features during peptide-spectrum matching and filtering, underscoring their practical importance in large-scale proteomic analyses. Protein Cleaver builds on this principle by offering a streamlined, structure-aware tool that performs *in silico* digestion and highlights regions of proteins that are more or less likely to yield detectable peptides based on mass and length constraints. We acknowledge that peptide detectability is a multifactorial concept, as established in foundational studies by Tang et al. (Tang et al., 2006) and Nesvizhskii and Aebersold, (2005), which introduced the idea that detectability is influenced by intrinsic peptide properties, sequence context, and experimental conditions. While Protein Cleaver does not currently incorporate machine learning-based detectability models, it is designed as a first-pass, interpretable tool that complements more complex predictive frameworks.

Collectively, our findings highlight the versatility and practical utility of Protein Cleaver in both targeted and large-scale proteomics analyses. By enabling comprehensive *in silico* digestion and structural visualization, Protein Cleaver is well-suited to support a wide array of proteomics applications. We anticipate that Protein Cleaver will serve as a valuable resource for the proteomics community, facilitating informed protease selection, enhancing peptide coverage, and ultimately contributing to the advancement of proteomic research.

## Data Availability

The original contributions presented in the study are included in the article/Supplementary Material, further inquiries can be directed to the corresponding author.
