# Supplementary material for: Protein cleaver: an interactive web interface for in silico prediction and systematic annotation of protein digestion-derived peptides
Source: Front Bioinform. 2025 Sep 4;5:1576317. doi: 10.3389/fbinf.2025.1576317 (PMC12445168; doi:10.3389/fbinf.2025.1576317)
Supplement: Supplementary file 1 [file Table1.docx]

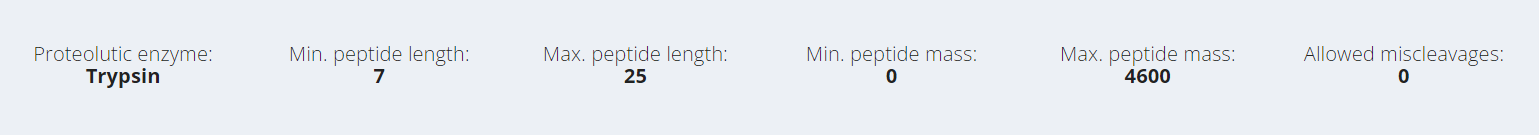


**Supplementary Table 1.** Human proteins likely to not be detected using trypsin and the specified parameters due to non-identifiable peptides. 114 in total.

Only reviewed proteins have been selected from UniProt including isoforms

| UniProtID | Gene name | Protein name | Identifiable peptides | Total peptides | Protein length | Max. coverage [%] |
| --- | --- | --- | --- | --- | --- | --- |
| A0A075B706 | TRDJ1 | T cell receptor delta joining 1 | 0 | 4 | 16 | 0 |
| A0A0A0MS00 | IGLV3-32 | Probable non-functional immunoglobulin lambda variable 3-32 | 0 | 5 | 114 | 0 |
| A0A411D538 | SCPPPQ1 | Secretory calcium-binding phosphoprotein proline-glutamine rich 1 | 0 | 3 | 79 | 0 |
| A1A580 | KRTAP23-1 | Keratin-associated protein 23-1 | 0 | 2 | 65 | 0 |
| A4D0T7 | SMIM30 | Small integral membrane protein 30 | 0 | 4 | 59 | 0 |
| C9JFL3 | PHGR1 | Proline, histidine and glycine-rich protein 1 | 0 | 2 | 82 | 0 |
| E0CX11 | STMP1 | Short transmembrane mitochondrial protein 1 | 0 | 7 | 47 | 0 |
| G5E9R7 | KRTAP4-16 | Putative keratin-associated protein 4-16 | 0 | 6 | 235 | 0 |
| O15225 | INE1 | Putative inactivation escape 1 protein | 0 | 1 | 51 | 0 |
| O15432 | SLC31A2 | Protein SLC31A2 | 0 | 6 | 143 | 0 |
| O75081-5 | CBFA2T3 | Isoform 4 of Protein CBFA2T3 | 0 | 7 | 78 | 0 |
| O75177-3 | SS18L1 | Isoform 3 of Calcium-responsive transactivator | 0 | 4 | 314 | 0 |
| O75177-4 | SS18L1 | Isoform 4 of Calcium-responsive transactivator | 0 | 4 | 265 | 0 |
| O75340-3 | PDCD6 | Isoform 3 of Programmed cell death protein 6 | 0 | 4 | 69 | 0 |
| O94777 | DPM2 | Dolichol phosphate-mannose biosynthesis regulatory protein | 0 | 8 | 84 | 0 |
| O95424 | DEXI | Dexamethasone-induced protein | 0 | 2 | 95 | 0 |
| P01358 | sp\|P01358\|GAJU_HUMAN | Gastric juice peptide 1 | 0 | 2 | 10 | 0 |
| P01858 | sp\|P01858\|TUFT_HUMAN | Phagocytosis-stimulating peptide | 0 | 1 | 4 | 0 |
| P02808-2 | STATH | Isoform 2 of Statherin | 0 | 2 | 52 | 0 |
| P02810 | PRH1 | Salivary acidic proline-rich phosphoprotein 1/2 | 0 | 3 | 166 | 0 |
| P02812 | PRB2 | Basic salivary proline-rich protein 2 | 0 | 15 | 416 | 0 |
| P04280 | PRB1 | Basic salivary proline-rich protein 1 | 0 | 12 | 392 | 0 |
| P04553 | PRM1 | Sperm protamine P1 | 0 | 17 | 51 | 0 |
| P09430 | TNP1 | Spermatid nuclear transition protein 1 | 0 | 22 | 55 | 0 |
| P0C5Y4 | KRTAP1-4 | Keratin-associated protein 1-4 | 0 | 3 | 121 | 0 |
| P0C6T2 | OST4 | Dolichyl-diphosphooligosaccharide--protein glycosyltransferase subunit 4 | 0 | 3 | 37 | 0 |
| P0DN84 | STRIT1 | Sarcoplasmic/endoplasmic reticulum calcium ATPase regulator DWORF | 0 | 2 | 35 | 0 |
| P0DOY5 | IGHD1-1 | Immunoglobulin heavy diversity 1-1 | 0 | 1 | 5 | 0 |
| P0DPI4 | TRBD1 | T cell receptor beta diversity 1 | 0 | 1 | 4 | 0 |
| P0DPR3 | TRDD1 | T cell receptor delta diversity 1 | 0 | 1 | 2 | 0 |
| P10163 | PRB4 | Basic salivary proline-rich protein 4 | 0 | 5 | 310 | 0 |
| P21145 | MAL | Myelin and lymphocyte protein | 0 | 4 | 153 | 0 |
| P21145-2 | MAL | Isoform B of Myelin and lymphocyte protein | 0 | 3 | 111 | 0 |
| P21145-3 | MAL | Isoform C of Myelin and lymphocyte protein | 0 | 4 | 97 | 0 |
| P21145-4 | MAL | Isoform D of Myelin and lymphocyte protein | 0 | 3 | 55 | 0 |
| P22103 | sp\|P22103\|PNEU_HUMAN | Pneumadin | 0 | 2 | 10 | 0 |
| P31358 | CD52 | CAMPATH-1 antigen | 0 | 3 | 61 | 0 |
| P35372-17 | OPRM1 | Isoform 17 of Mu-type opioid receptor | 0 | 3 | 101 | 0 |
| P49447-2 | CYB561 | Isoform 2 of Transmembrane ascorbate-dependent reductase CYB561 | 0 | 10 | 138 | 0 |
| P59052-2 | B3GALT5-AS1 | Isoform 2 of Putative uncharacterized protein B3GALT5-AS1 | 0 | 9 | 64 | 0 |
| P59990 | KRTAP12-1 | Keratin-associated protein 12-1 | 0 | 2 | 96 | 0 |
| P59991 | KRTAP12-2 | Keratin-associated protein 12-2 | 0 | 2 | 146 | 0 |
| P60328 | KRTAP12-3 | Keratin-associated protein 12-3 | 0 | 2 | 96 | 0 |
| P60329 | KRTAP12-4 | Keratin-associated protein 12-4 | 0 | 1 | 112 | 0 |
| P60331 | KRTAP10-1 | Keratin-associated protein 10-1 | 0 | 4 | 282 | 0 |
| P60368 | KRTAP10-2 | Keratin-associated protein 10-2 | 0 | 5 | 255 | 0 |
| P60896 | SEM1 | 26S proteasome complex subunit SEM1 | 0 | 6 | 70 | 0 |
| P62945 | RPL41 | Small ribosomal subunit protein eS32 | 0 | 17 | 25 | 0 |
| P62952 | BLCAP | Apoptosis inducing factor BLCAP | 0 | 2 | 87 | 0 |
| Q07326 | PIGF | Phosphatidylinositol-glycan biosynthesis class F protein | 0 | 14 | 219 | 0 |
| Q0D2K3-2 | RIPPLY1 | Isoform 2 of Protein ripply1 | 0 | 4 | 104 | 0 |
| Q13021 | MALL | MAL-like protein | 0 | 6 | 153 | 0 |
| Q156A1 | ATXN8 | Ataxin-8 | 0 | 1 | 80 | 0 |
| Q16617 | NKG7 | Protein NKG7 | 0 | 3 | 165 | 0 |
| Q1W4C9-2 | SPINK13 | Isoform 2 of Serine protease inhibitor Kazal-type 13 | 0 | 6 | 54 | 0 |
| Q2I0M4-2 | LRRC26 | Isoform 2 of Leucine-rich repeat-containing protein 26 | 0 | 5 | 45 | 0 |
| Q3LI54 | KRTAP19-8 | Keratin-associated protein 19-8 | 0 | 4 | 63 | 0 |
| Q3LI58 | KRTAP21-1 | Keratin-associated protein 21-1 | 0 | 3 | 79 | 0 |
| Q3LI68 | KRTAP22-2 | Keratin-associated protein 22-2 | 0 | 5 | 45 | 0 |
| Q3MUY2 | PIGY | Phosphatidylinositol N-acetylglucosaminyltransferase subunit Y | 0 | 3 | 71 | 0 |
| Q495B1-6 | ANKDD1A | Isoform 3 of Ankyrin repeat and death domain-containing protein 1A | 0 | 3 | 41 | 0 |
| Q4VC39 | HIGD2B | Putative HIG1 domain family member 2B | 0 | 8 | 106 | 0 |
| Q5T4I8-2 | C6orf52 | Isoform 2 of Putative uncharacterized protein C6orf52 | 0 | 3 | 65 | 0 |
| Q6NVV0 | MKRN9P | Putative makorin-5 | 0 | 1 | 33 | 0 |
| Q6P5S7-2 | RNASEK | Isoform 2 of Ribonuclease kappa | 0 | 9 | 134 | 0 |
| Q6UWW9 | TMEM207 | Transmembrane protein 207 | 0 | 8 | 146 | 0 |
| Q6XCG6 | PP632 | Putative uncharacterized protein PP632 | 0 | 6 | 107 | 0 |
| Q6ZP80-3 | TMEM182 | Isoform 3 of Transmembrane protein 182 | 0 | 5 | 110 | 0 |
| Q71RG4-4 | TMUB2 | Isoform 4 of Transmembrane and ubiquitin-like domain-containing protein 2 | 0 | 3 | 94 | 0 |
| Q7Z429 | GRINA | Protein lifeguard 1 | 0 | 17 | 371 | 0 |
| Q7Z4W3 | KRTAP19-3 | Keratin-associated protein 19-3 | 0 | 3 | 81 | 0 |
| Q8IUB9 | KRTAP19-1 | Keratin-associated protein 19-1 | 0 | 3 | 90 | 0 |
| Q8IVK1 | GLYCAM1 | Putative glycosylation-dependent cell adhesion molecule 1 | 0 | 5 | 47 | 0 |
| Q8IVK1-2 | GLYCAM1 | Isoform 2 of Putative glycosylation-dependent cell adhesion molecule 1 | 0 | 2 | 33 | 0 |
| Q8IXM6-6 | NRM | Isoform 3 of Nurim | 0 | 4 | 109 | 0 |
| Q8IZ96-16 | CMTM1 | Isoform 19 of CKLF-like MARVEL transmembrane domain-containing protein 1 | 0 | 6 | 71 | 0 |
| Q8IZV2 | CMTM8 | CKLF-like MARVEL transmembrane domain-containing protein 8 | 0 | 10 | 173 | 0 |
| Q8IZV2-2 | CMTM8 | Isoform 2 of CKLF-like MARVEL transmembrane domain-containing protein 8 | 0 | 8 | 115 | 0 |
| Q8N114-3 | SHISA5 | Isoform 3 of Protein shisa-5 | 0 | 4 | 137 | 0 |
| Q8N114-4 | SHISA5 | Isoform 4 of Protein shisa-5 | 0 | 3 | 118 | 0 |
| Q8N688-2 | DEFB123 | Isoform 2 of Beta-defensin 123 | 0 | 2 | 28 | 0 |
| Q8N6Y0-2 | USHBP1 | Isoform 2 of Harmonin-binding protein USHBP1 | 0 | 6 | 68 | 0 |
| Q8N8F6 | YIPF7 | Protein YIPF7 | 0 | 6 | 256 | 0 |
| Q8N8F6-3 | YIPF7 | Isoform 3 of Protein YIPF7 | 0 | 3 | 44 | 0 |
| Q8NHV5-3 | MOSMO | Isoform 2 of Modulator of smoothened protein | 0 | 2 | 38 | 0 |
| Q8NHW4-6 | CCL4L1 | Isoform 6 of C-C motif chemokine 4-like | 0 | 9 | 86 | 0 |
| Q8TED4-4 | SLC37A2 | Isoform 4 of Glucose-6-phosphate exchanger SLC37A2 | 0 | 9 | 126 | 0 |
| Q8WY50 | PLAC4 | Placenta-specific protein 4 | 0 | 6 | 150 | 0 |
| Q96K37-3 | SLC35E1 | Isoform 3 of Solute carrier family 35 member E1 | 0 | 9 | 104 | 0 |
| Q96T76-6 | MMS19 | Isoform 3 of MMS19 nucleotide excision repair protein homolog | 0 | 1 | 40 | 0 |
| Q99217 | AMELX | Amelogenin, X isoform | 0 | 5 | 191 | 0 |
| Q99218-1 | AMELY | Isoform 1 of Amelogenin, Y isoform | 0 | 6 | 192 | 0 |
| Q9BQ66 | KRTAP4-12 | Keratin-associated protein 4-12 | 0 | 5 | 201 | 0 |
| Q9BQY9-3 | DBNDD2 | Isoform 3 of Dysbindin domain-containing protein 2 | 0 | 8 | 112 | 0 |
| Q9BWQ8-2 | FAIM2 | Isoform 2 of Protein lifeguard 2 | 0 | 12 | 270 | 0 |
| Q9BY19 | MS4A8 | Membrane-spanning 4-domains subfamily A member 8 | 0 | 4 | 250 | 0 |
| Q9BYP8 | KRTAP17-1 | Keratin-associated protein 17-1 | 0 | 1 | 105 | 0 |
| Q9BYQ5 | KRTAP4-6 | Keratin-associated protein 4-6 | 0 | 6 | 205 | 0 |
| Q9BYQ8 | KRTAP4-9 | Keratin-associated protein 4-9 | 0 | 5 | 210 | 0 |
| Q9BYQ9 | KRTAP4-8 | Keratin-associated protein 4-8 | 0 | 3 | 185 | 0 |
| Q9BYR0 | KRTAP4-7 | Keratin-associated protein 4-7 | 0 | 4 | 155 | 0 |
| Q9BYR2 | KRTAP4-5 | Keratin-associated protein 4-5 | 0 | 5 | 181 | 0 |
| Q9BZ97 | TTTY13 | Putative transcript Y 13 protein | 0 | 3 | 58 | 0 |
| Q9HC47 | CTAGE1 | Cutaneous T-cell lymphoma-associated antigen 1 | 0 | 1 | 74 | 0 |
| Q9NPH6-3 | OBP2B | Isoform Bg of Odorant-binding protein 2b | 0 | 4 | 85 | 0 |
| Q9NPU4 | C14orf132 | Uncharacterized protein C14orf132 | 0 | 2 | 83 | 0 |
| Q9NRI6 | PYY2 | Putative peptide YY-2 | 0 | 1 | 33 | 0 |
| Q9NZF1 | PLAC8 | Placenta-specific gene 8 protein | 0 | 9 | 115 | 0 |
| Q9NZM6-3 | PKD2L2 | Isoform 3 of Polycystin-2-like protein 2 | 0 | 7 | 57 | 0 |
| Q9P0N5 | TMEM216 | Transmembrane protein 216 | 0 | 13 | 145 | 0 |
| Q9P0N5-3 | TMEM216 | Isoform 3 of Transmembrane protein 216 | 0 | 13 | 148 | 0 |
| Q9UBC7-2 | GALP | Isoform 2 of Galanin-like peptide | 0 | 7 | 49 | 0 |
| Q9UKJ3-3 | GPATCH8 | Isoform 3 of G patch domain-containing protein 8 | 0 | 4 | 43 | 0 |
| Q9Y6X1 | SERP1 | Stress-associated endoplasmic reticulum protein 1 | 0 | 11 | 66 | 0 |


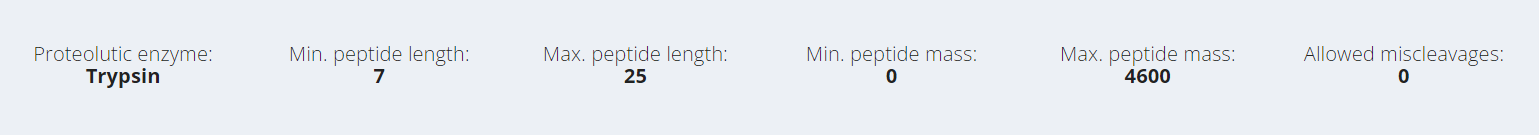
 **Supplementary Table 2.** Yeast proteins likely to not be detected using trypsin and the specified parameters due to non-identifiable peptides. 19 proteins in total.

Only reviewed proteins have been selected from UniProt including isoforms

| UniProtID | Gene name | Protein name | Identifiable peptides | Total peptides | | Protein length | Max. coverage [%] |
| --- | --- | --- | --- | --- | --- | --- | --- |
| I2HB52 | YBR056W-A | Uncharacterized protein YBR056W-A | 0 | | 3 | 66 | 0 |
| O13512 | YAL064W-B | Uncharacterized membrane protein YAL064W-B | 0 | | 1 | 126 | 0 |
| P00856 | ATP8 | ATP synthase protein 8 | 0 | | 4 | 48 | 0 |
| P0C289 | YDR034C-A | Putative uncharacterized protein YDR034C-A | 0 | | 1 | 58 | 0 |
| P0CX86 | RPL41A | Small ribosomal subunit protein eS32A | 0 | | 17 | 25 | 0 |
| P0CX87 | RPL41B | Small ribosomal subunit protein eS32B | 0 | | 17 | 25 | 0 |
| P32903 | PMP1 | Plasma membrane ATPase proteolipid 1 | 0 | | 6 | 40 | 0 |
| P39977 | YEL068C | Uncharacterized protein YEL068C | 0 | | 13 | 110 | 0 |
| P40975 | PMP2 | Plasma membrane ATPase proteolipid 2 | 0 | | 6 | 43 | 0 |
| P89113 | DDR2 | Protein DDR2 | 0 | | 2 | 61 | 0 |
| Q02598 | YIL014C-A | Uncharacterized protein YIL014C-A | 0 | | 1 | 104 | 0 |
| Q07990 | YLR042C | Cell wall protein YLR042C | 0 | | 6 | 161 | 0 |
| Q2V2P5 | YIL102C-A | Uncharacterized protein YIL102C-A | 0 | | 7 | 75 | 0 |
| Q2V2Q2 | YCL048W-A | Uncharacterized protein YCL048W-A | 0 | | 2 | 79 | 0 |
| Q3E752 | SPO24 | Sporulation protein 24 | 0 | | 6 | 67 | 0 |
| Q8TGJ0 | YOR394C-A | Uncharacterized protein YOR394C-A | 0 | | 2 | 55 | 0 |
| Q8TGJ7 | YLL066W-B | Uncharacterized protein YLL066W-B | 0 | | 1 | 56 | 0 |
| Q8TGQ7 | YPR159C-A | Uncharacterized protein YPR159C-A | 0 | | 1 | 33 | 0 |
| Q99380 | OST4 | Dolichyl-diphosphooligosaccharide--protein glycosyltransferase subunit OST4 | 0 | | 2 | 36 | 0 |
