# Supplementary material for: Protein cleaver: an interactive web interface for in silico prediction and systematic annotation of protein digestion-derived peptides
Source: Front Bioinform. 2025 Sep 4;5:1576317. doi: 10.3389/fbinf.2025.1576317 (PMC12445168; doi:10.3389/fbinf.2025.1576317)
Supplement: Supplementary file 2 [file Presentation1.pptx]

## Slide 1
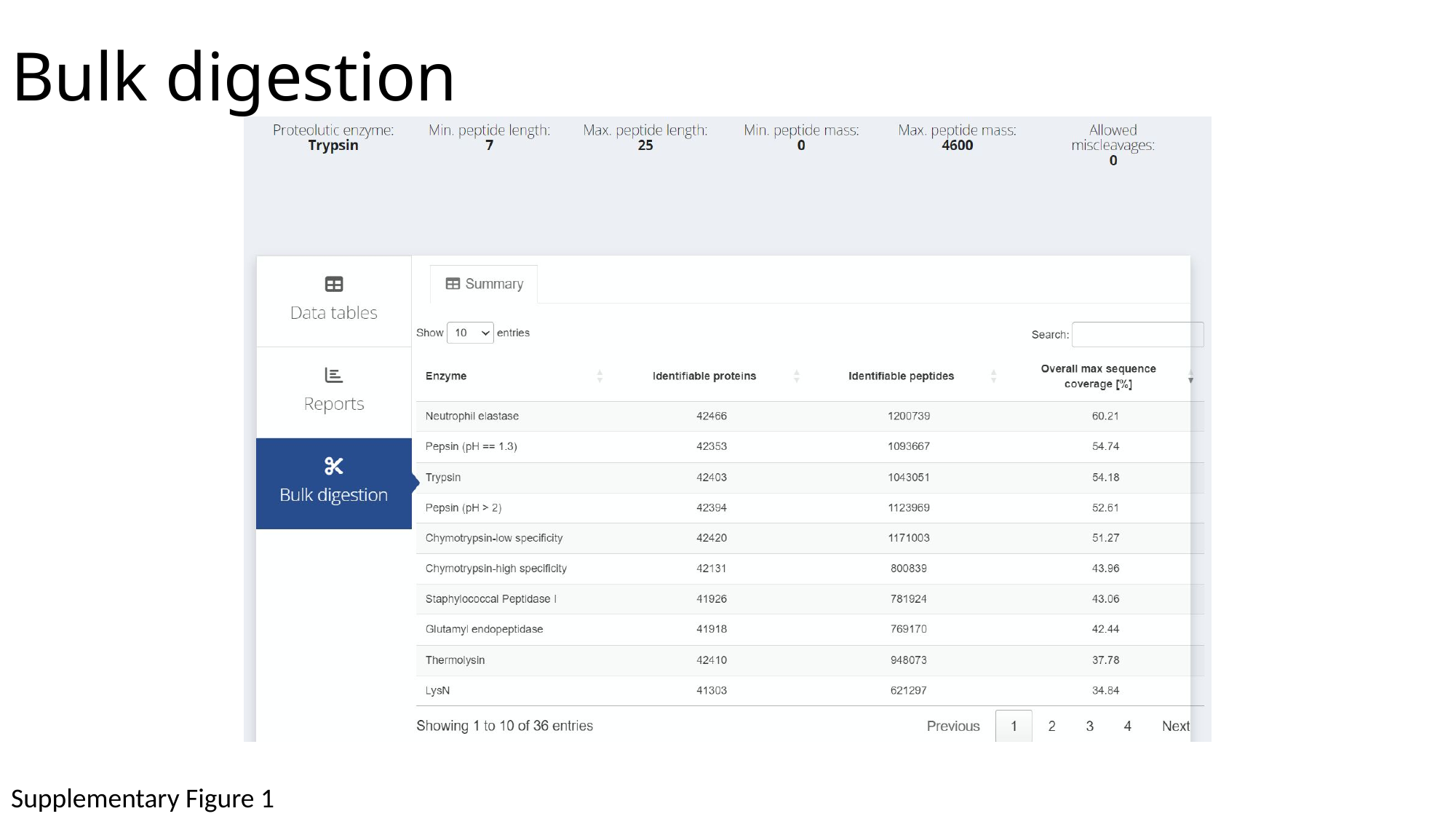

# Bulk digestion
Supplementary Figure 1

## Slide 2
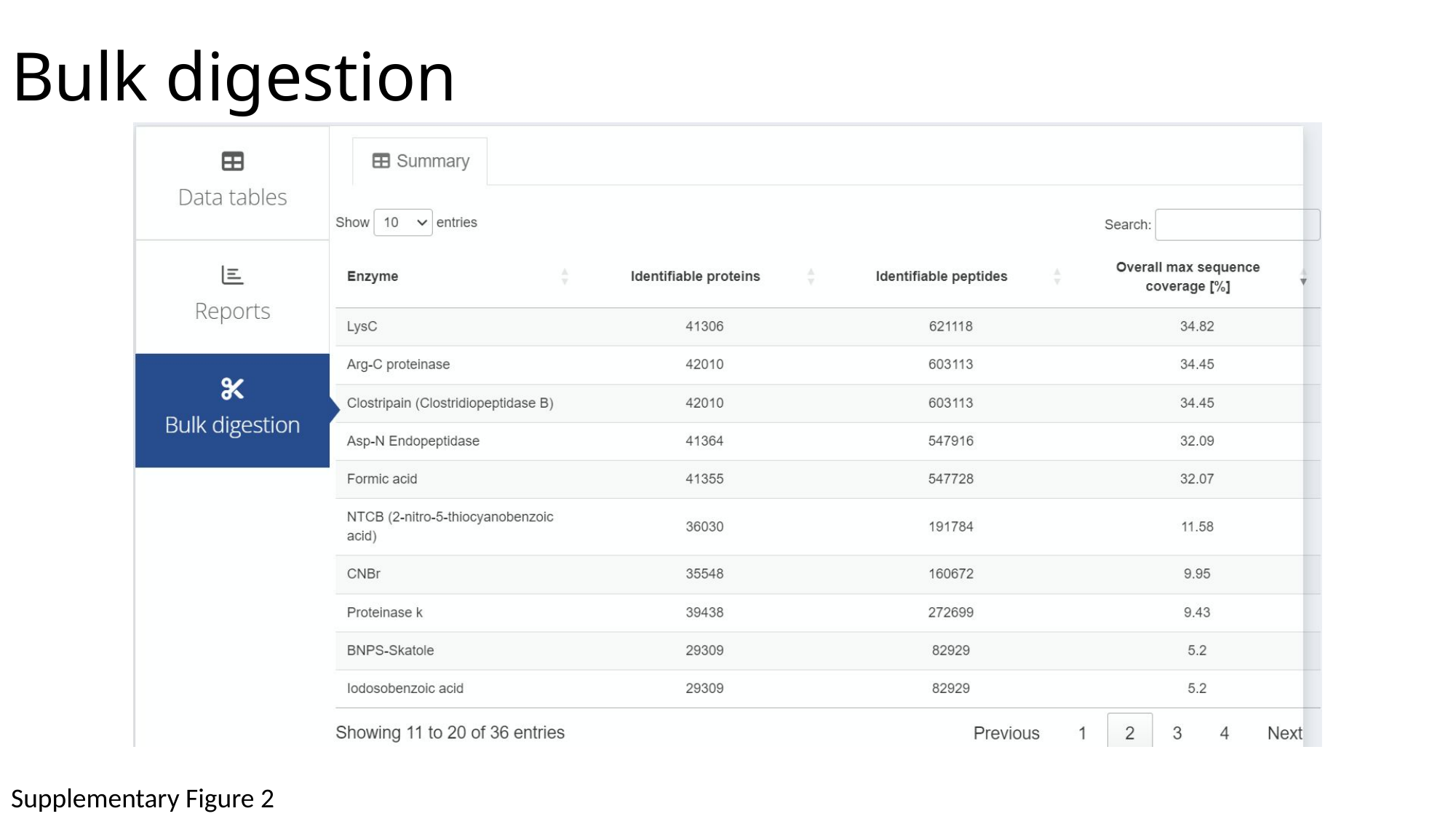

# Bulk digestion
Supplementary Figure 2

## Slide 3
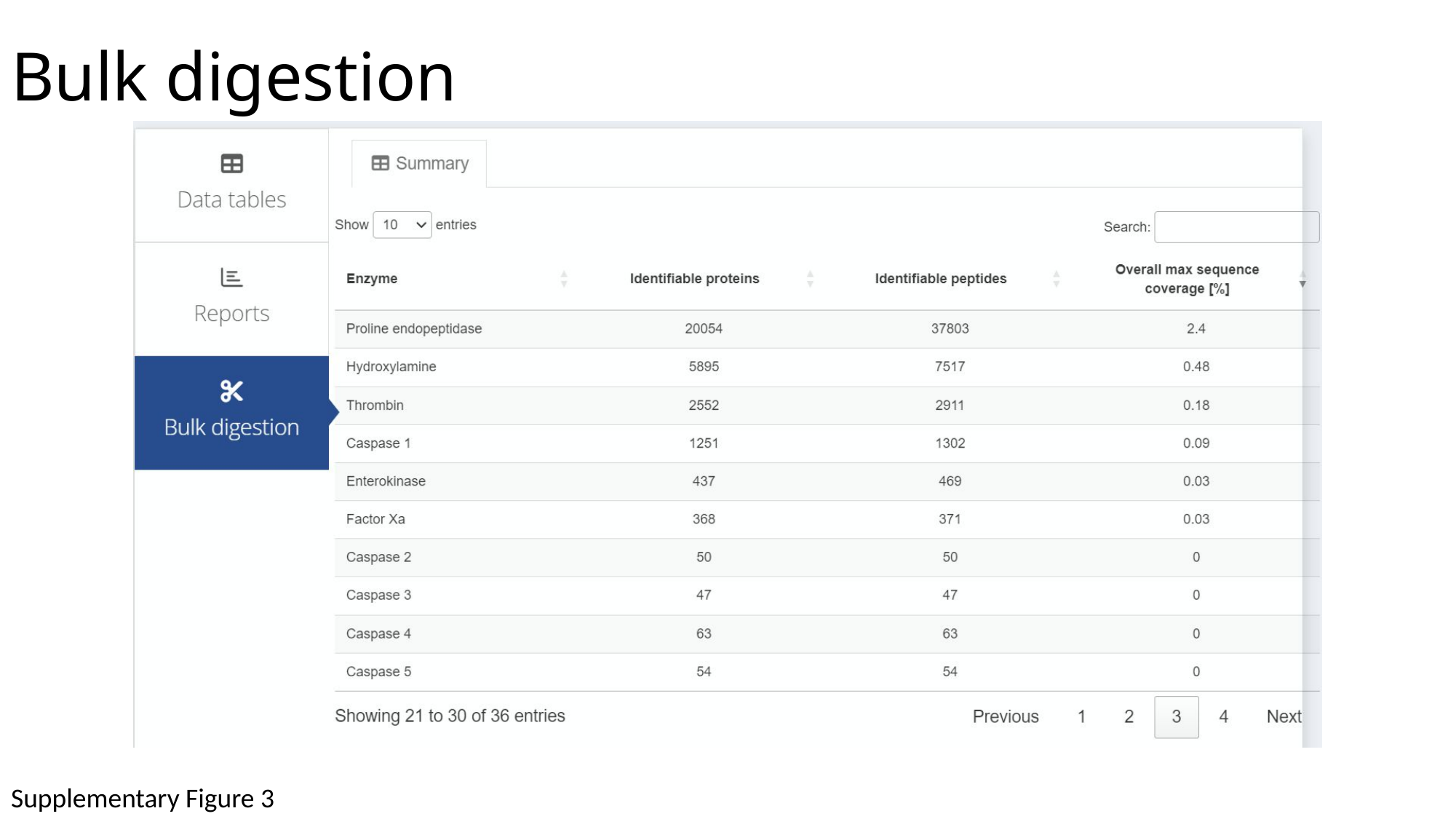

# Bulk digestion
Supplementary Figure 3

## Slide 4
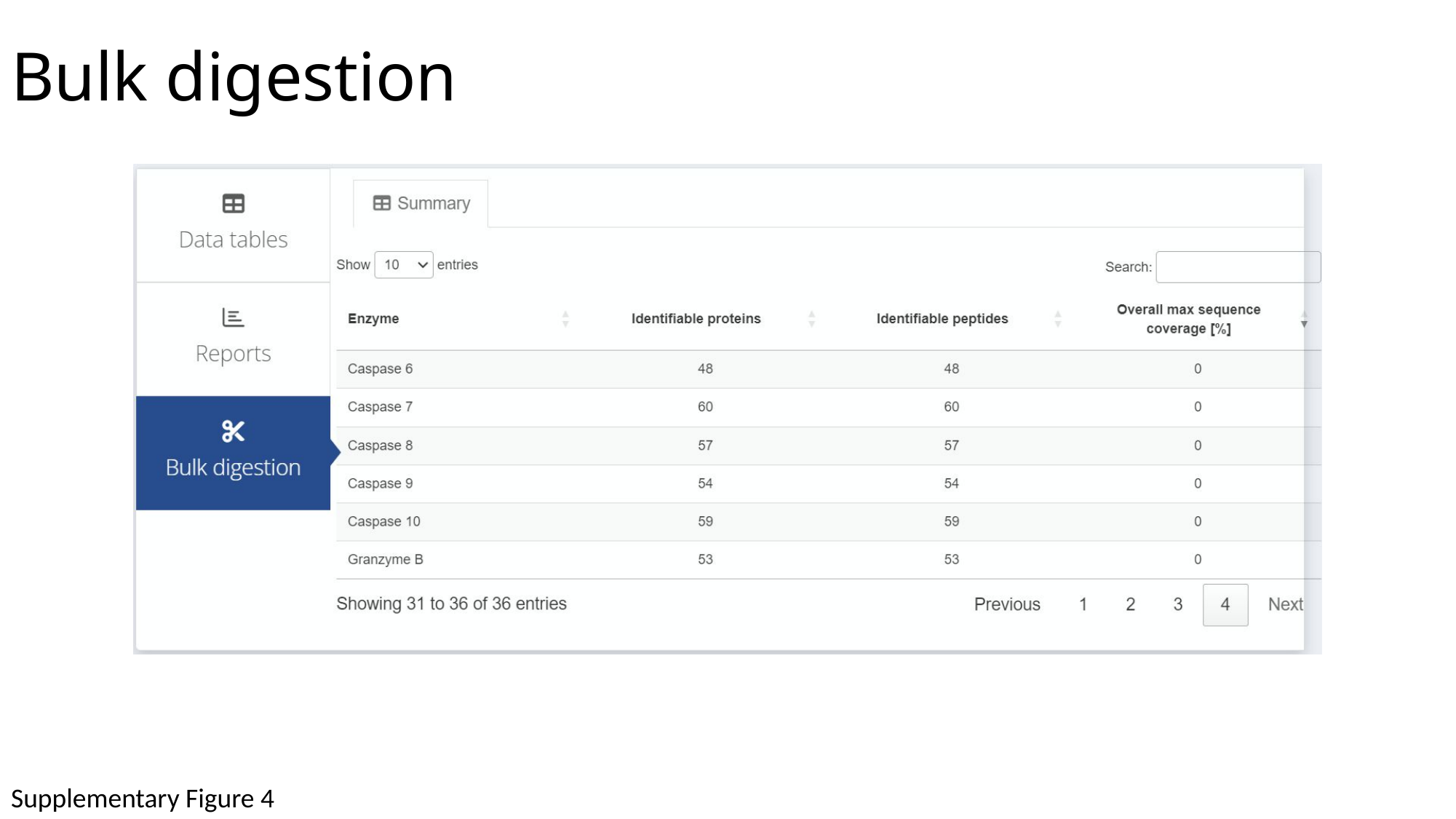

# Bulk digestion
Supplementary Figure 4

## Slide 5
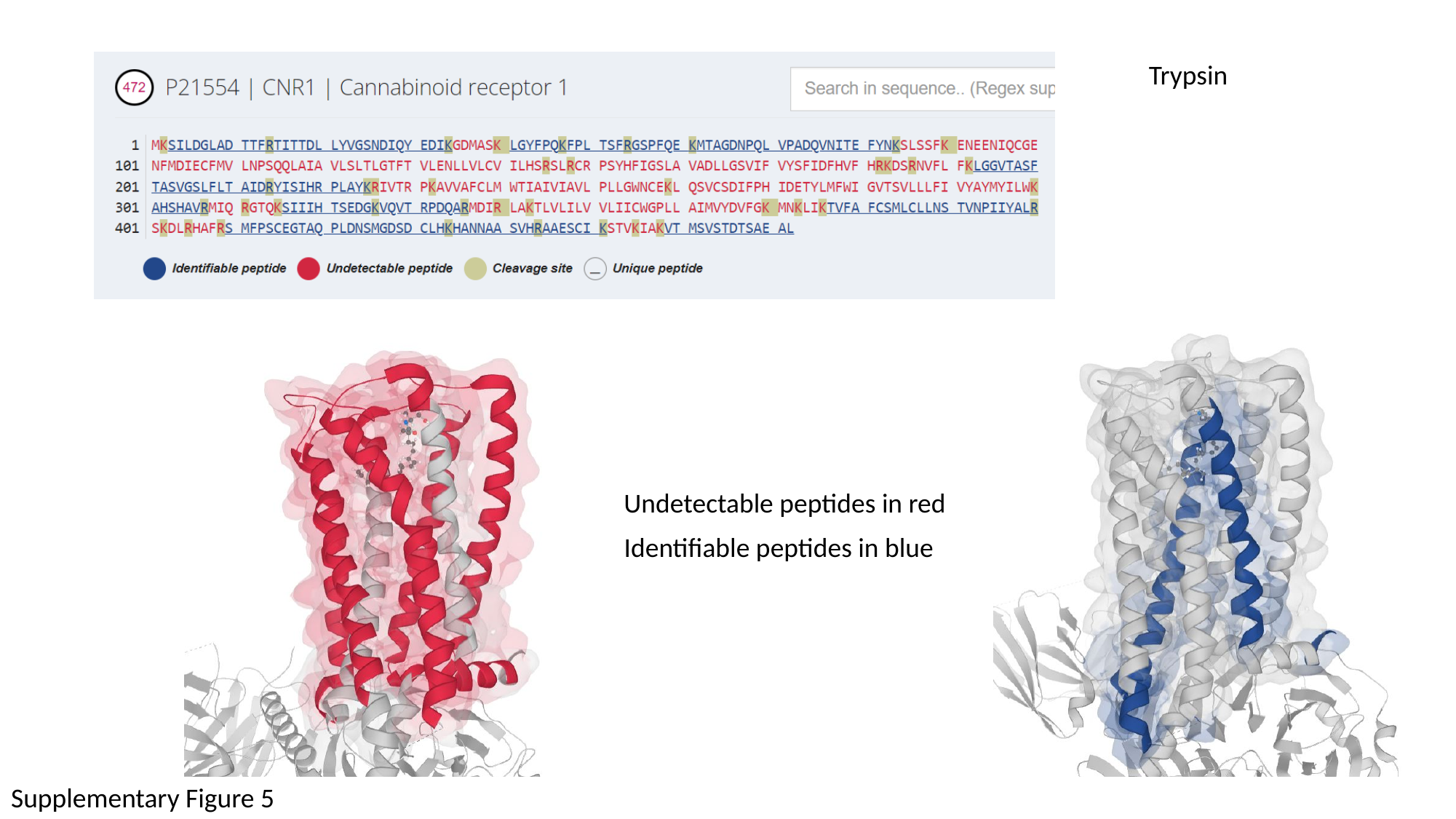

Trypsin
Undetectable peptides in red
Identifiable peptides in blue
Supplementary Figure 5

## Slide 6
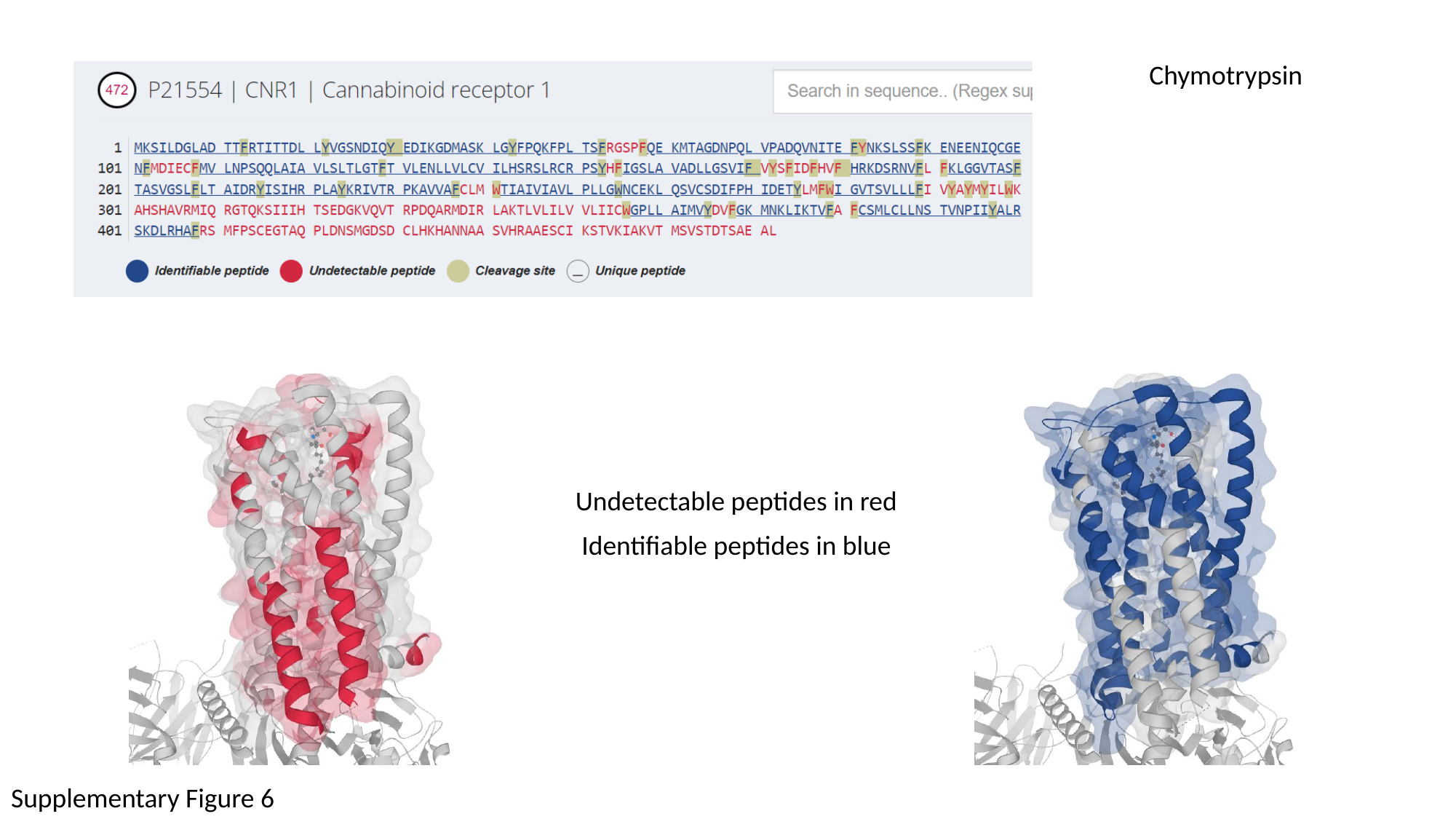

Chymotrypsin
Undetectable peptides in red
Identifiable peptides in blue
Supplementary Figure 6

## Slide 7
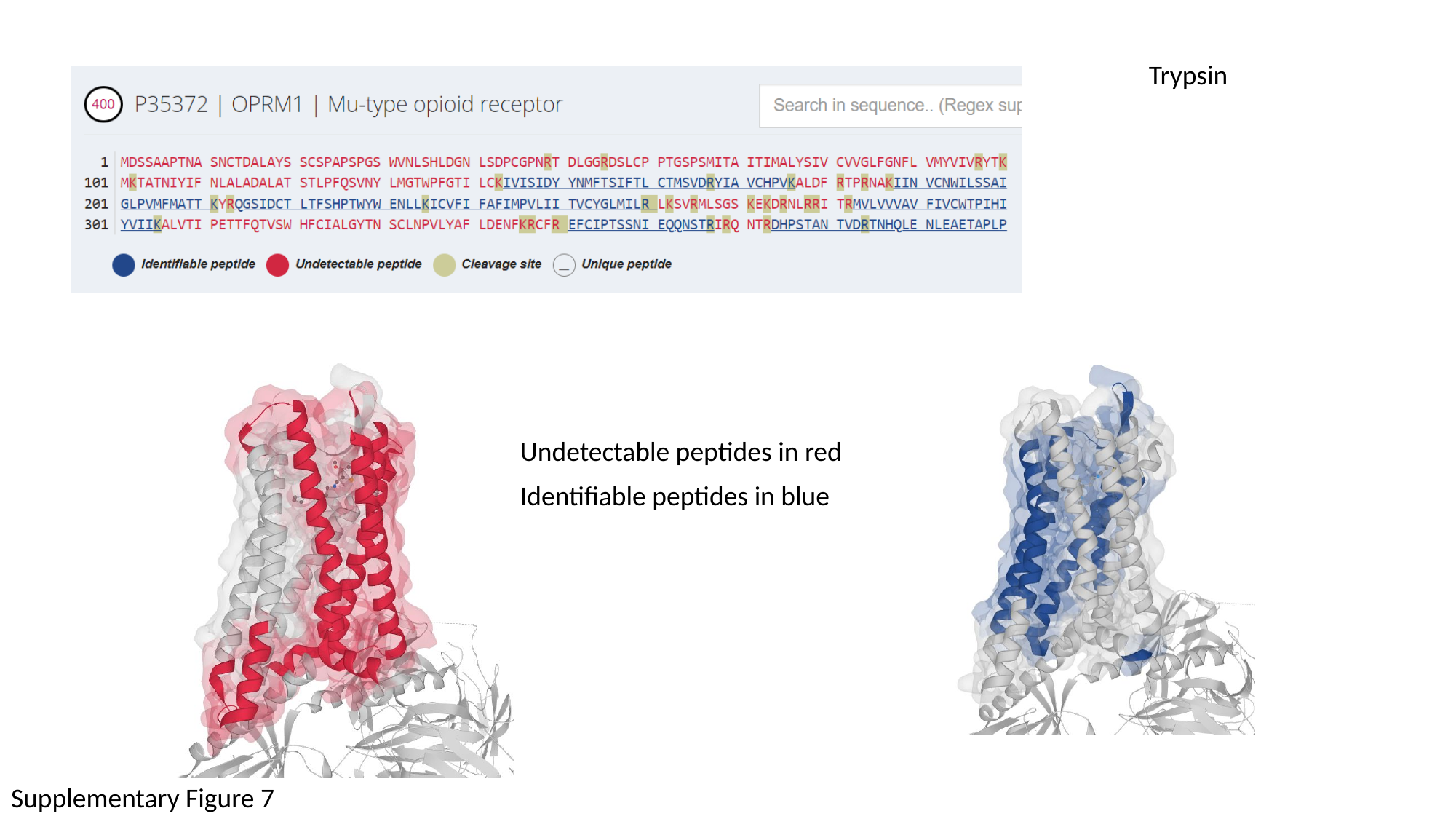

Trypsin
Undetectable peptides in red
Identifiable peptides in blue
Supplementary Figure 7

## Slide 8
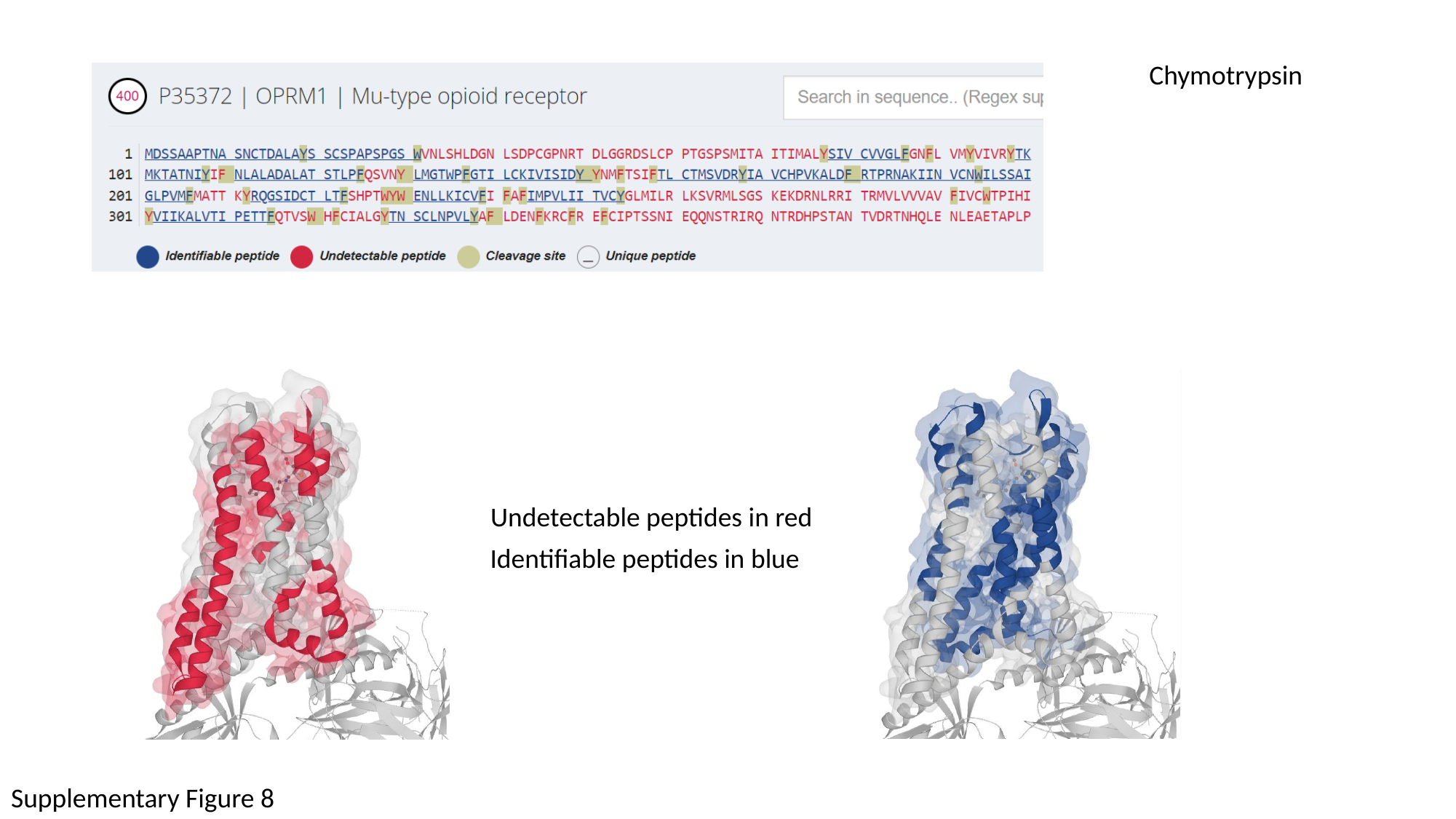

Chymotrypsin
Undetectable peptides in red
Identifiable peptides in blue
Supplementary Figure 8

## Slide 9
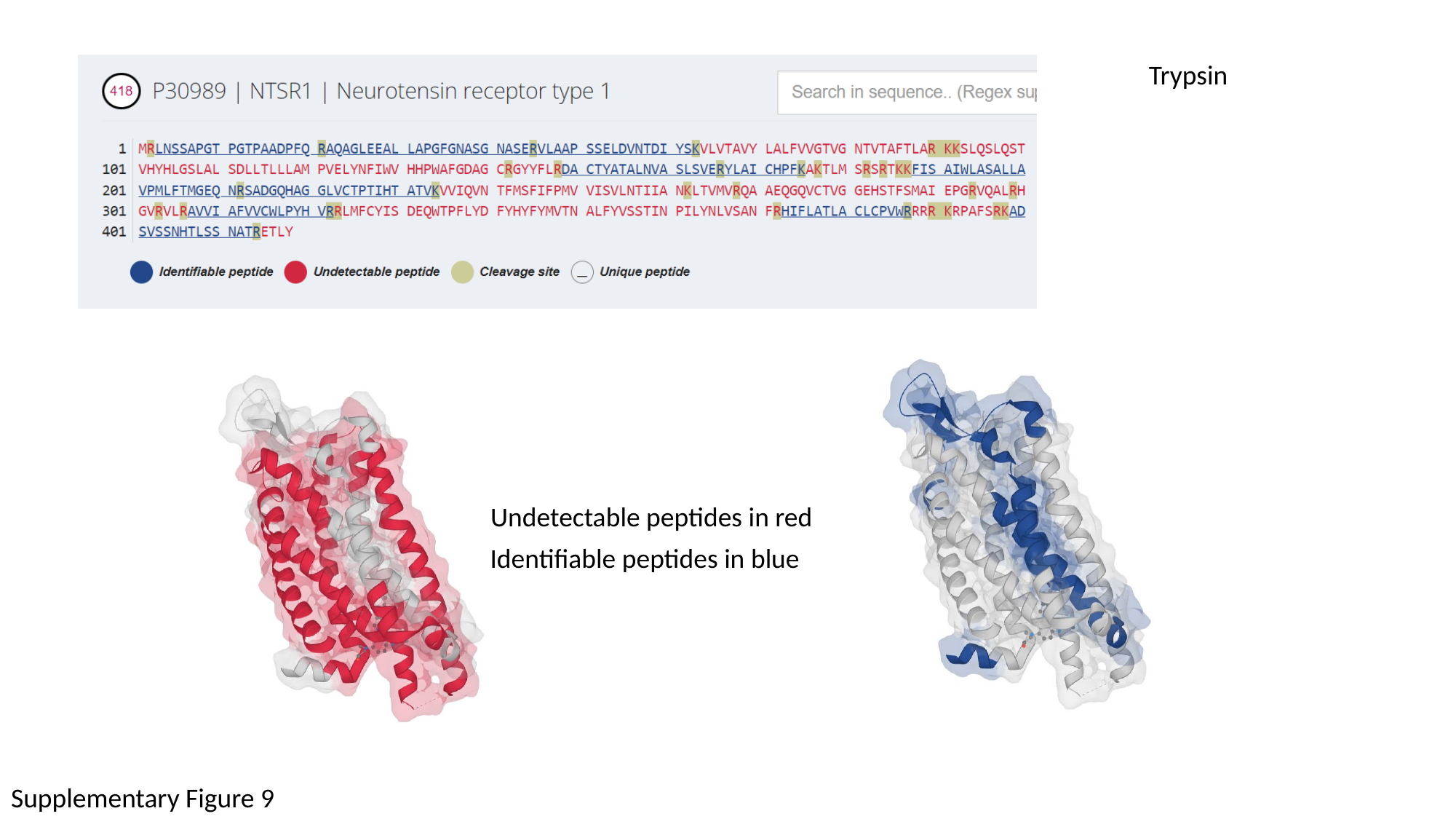

Trypsin
Undetectable peptides in red
Identifiable peptides in blue
Supplementary Figure 9

## Slide 10
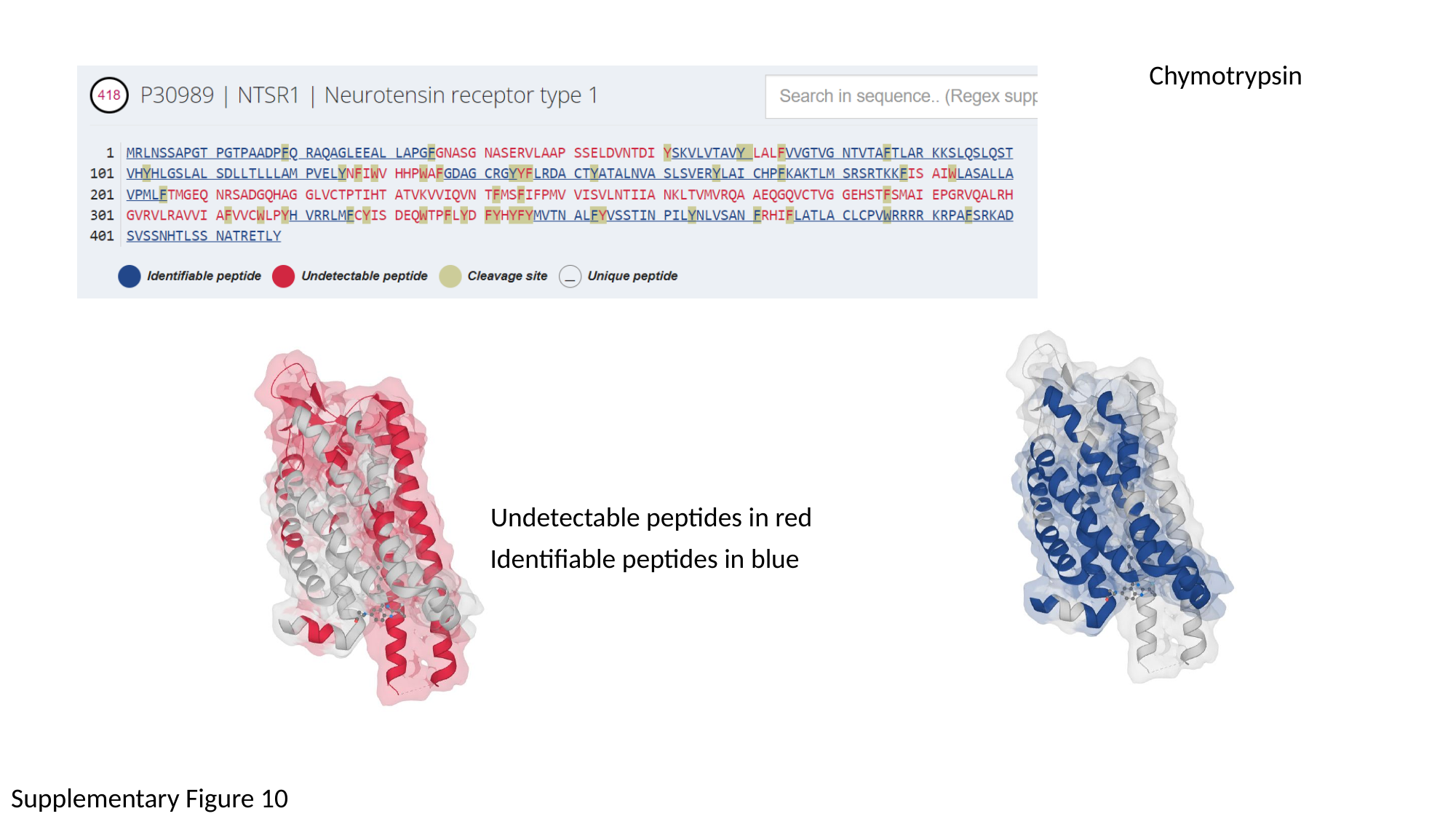

Chymotrypsin
Undetectable peptides in red
Identifiable peptides in blue
Supplementary Figure 10

## Slide 11
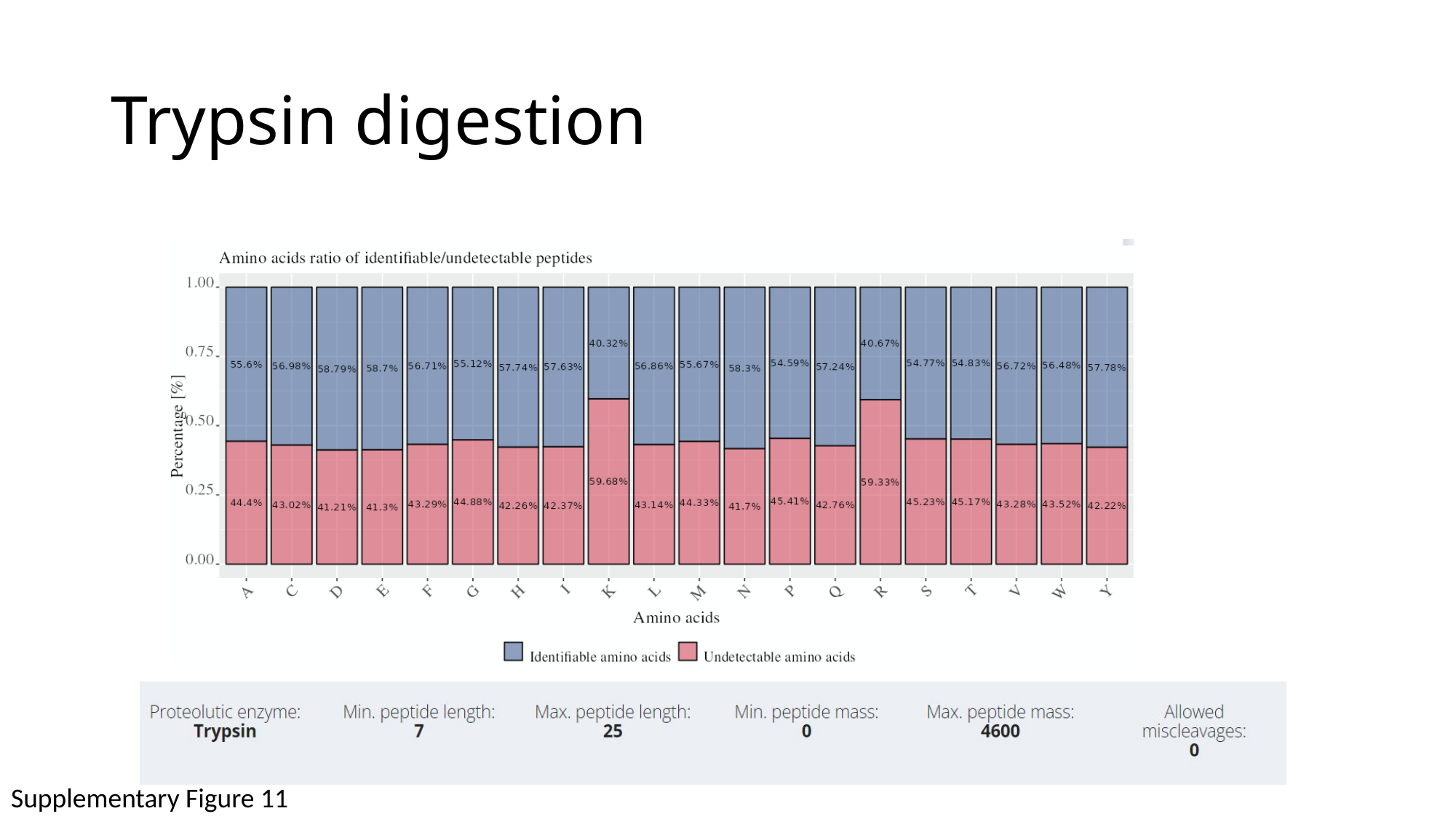

# Trypsin digestion
Supplementary Figure 11

## Slide 12
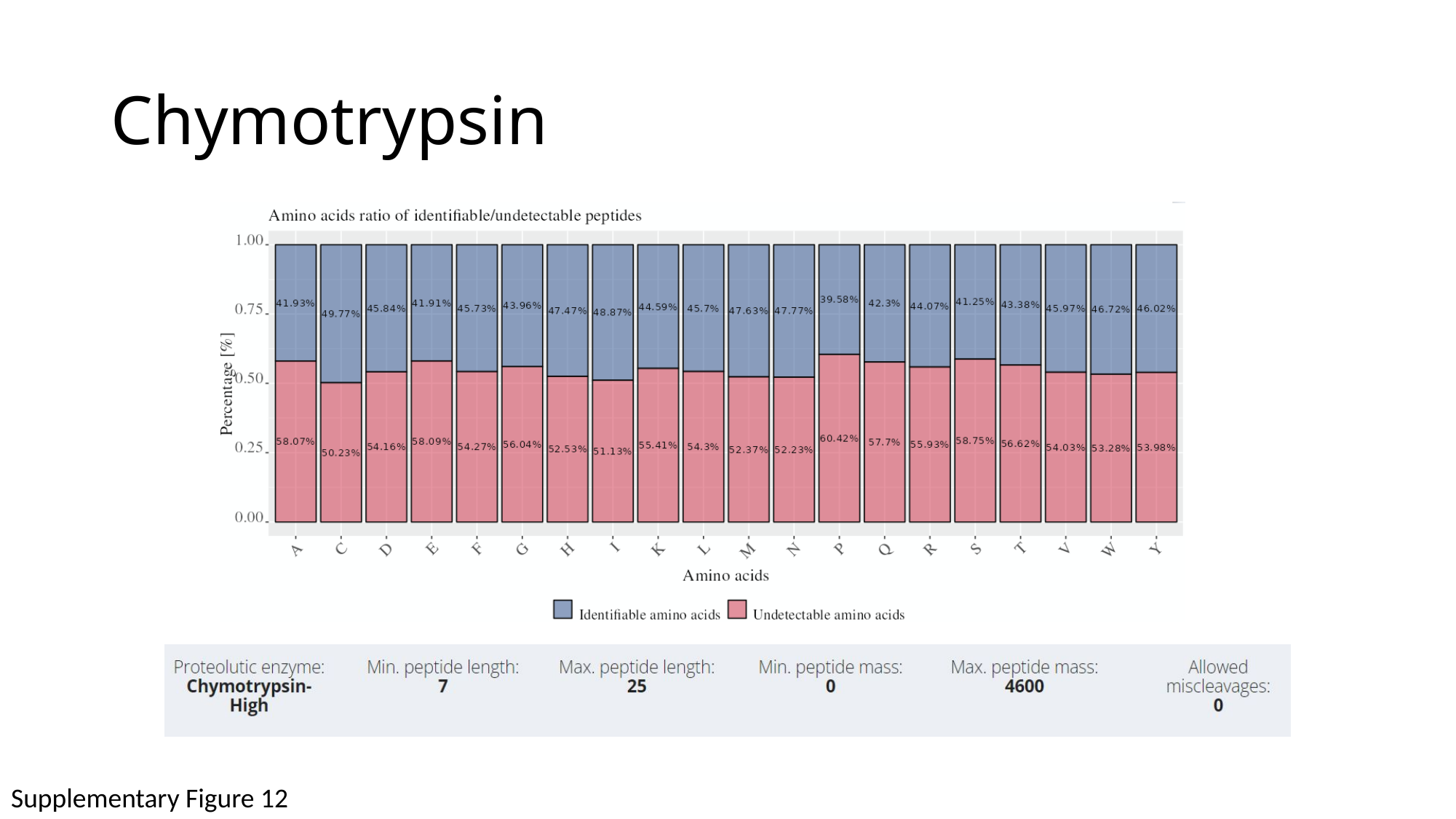

# Chymotrypsin
Supplementary Figure 12

## Slide 13
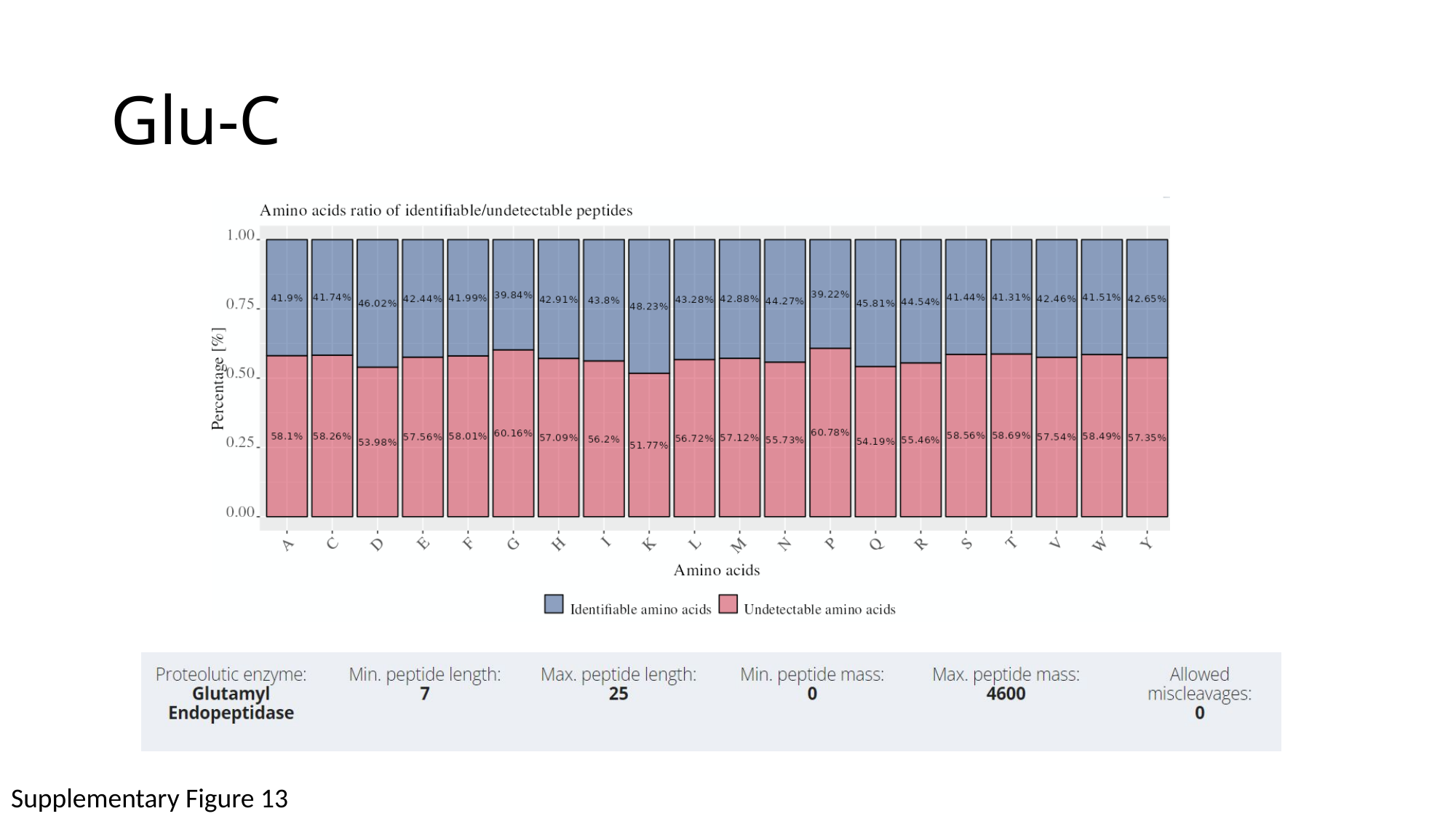

# Glu-C
Supplementary Figure 13

## Slide 14
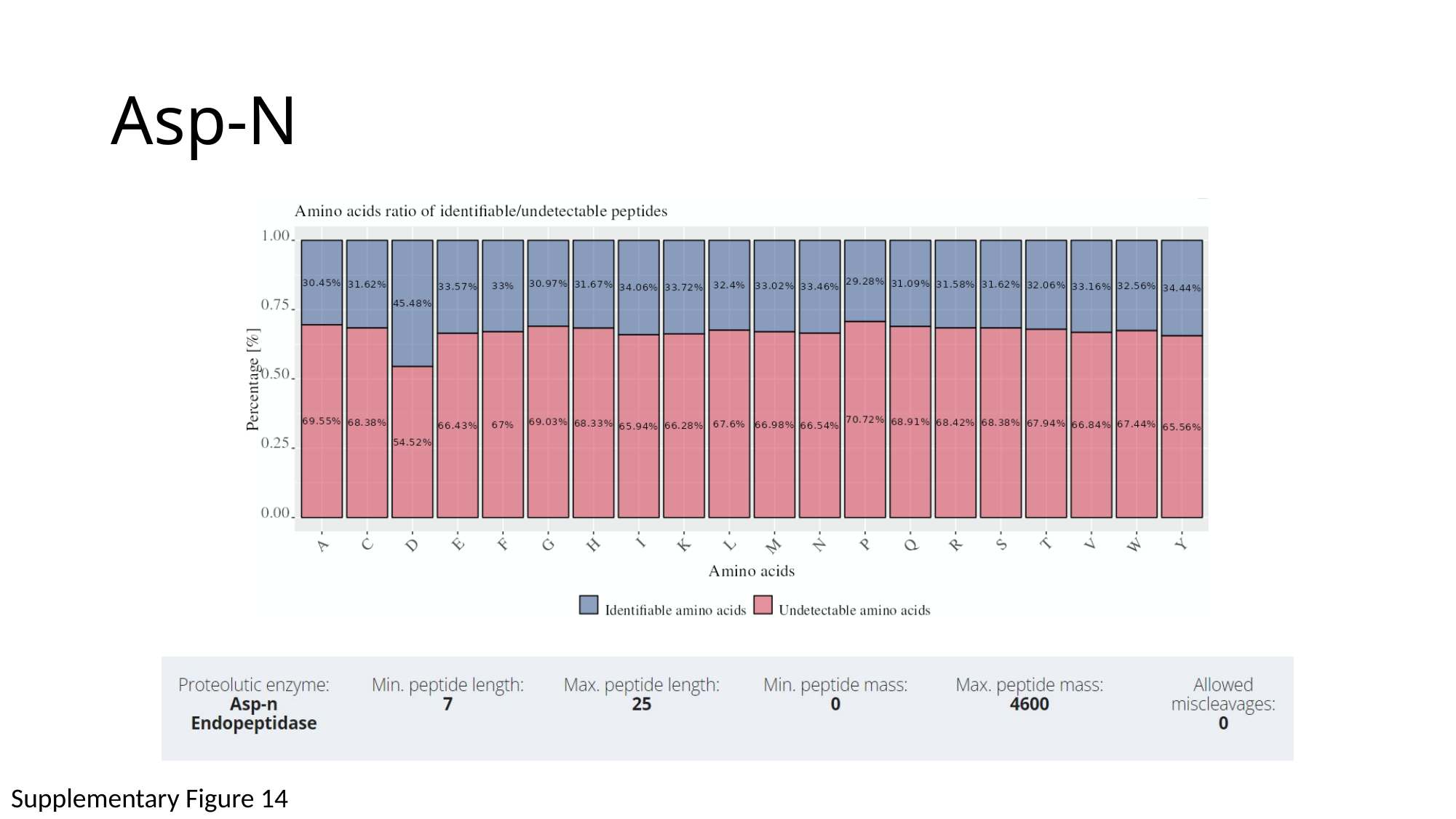

# Asp-N
Supplementary Figure 14

## Slide 15
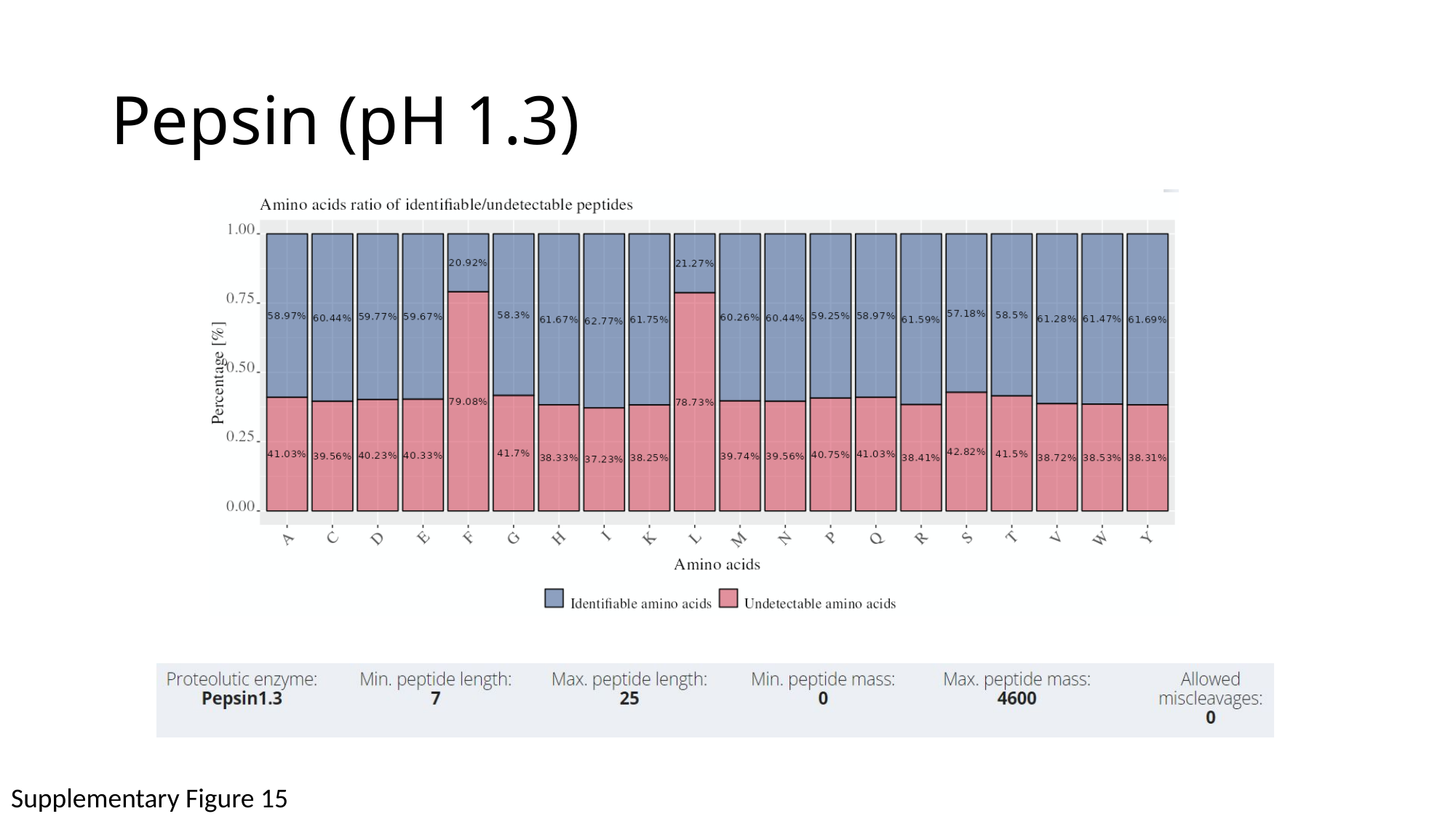

# Pepsin (pH 1.3)
Supplementary Figure 15

## Slide 16
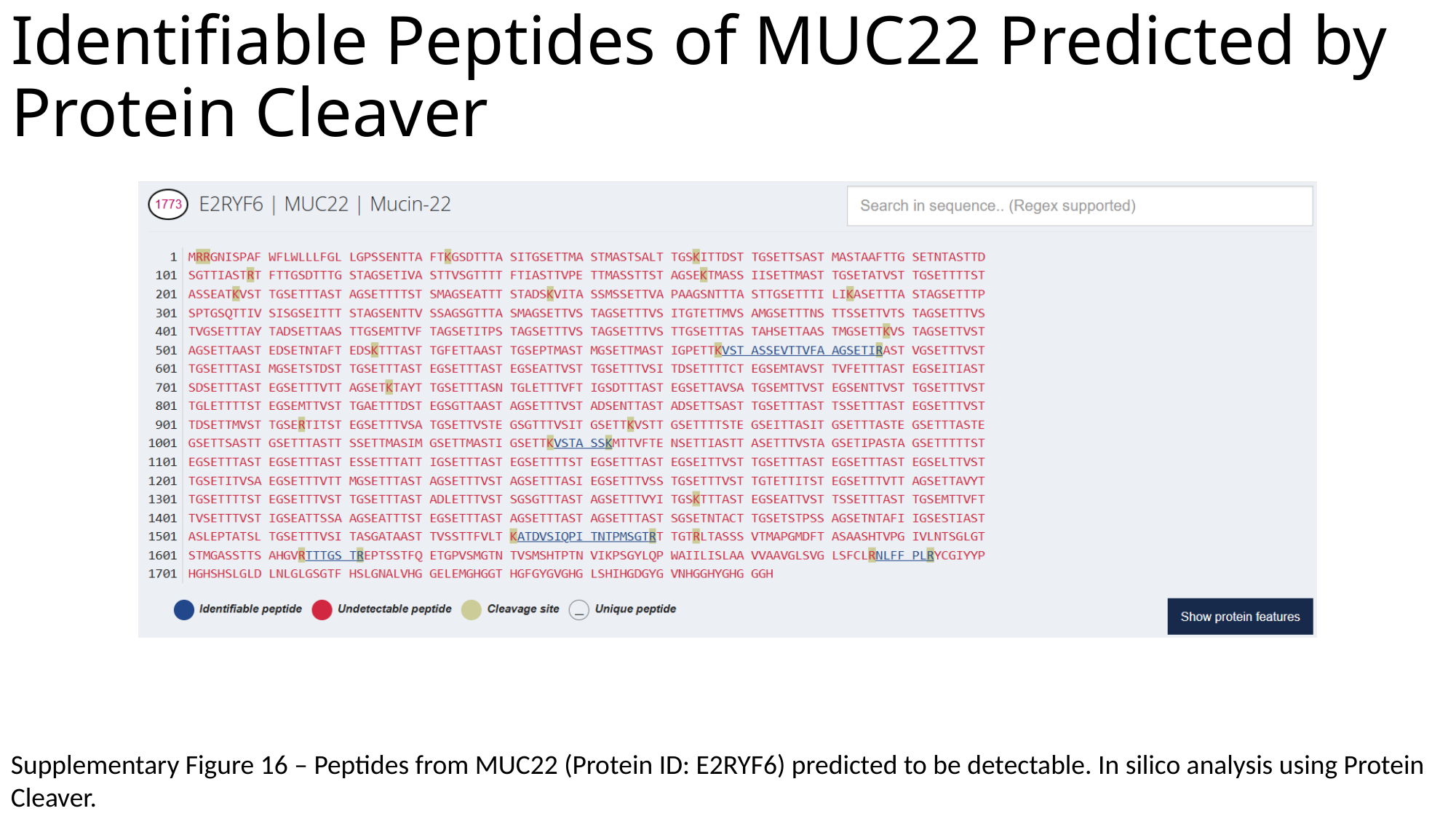

# Identifiable Peptides of MUC22 Predicted by Protein Cleaver
Supplementary Figure 16 – Peptides from MUC22 (Protein ID: E2RYF6) predicted to be detectable. In silico analysis using Protein Cleaver.

## Slide 17
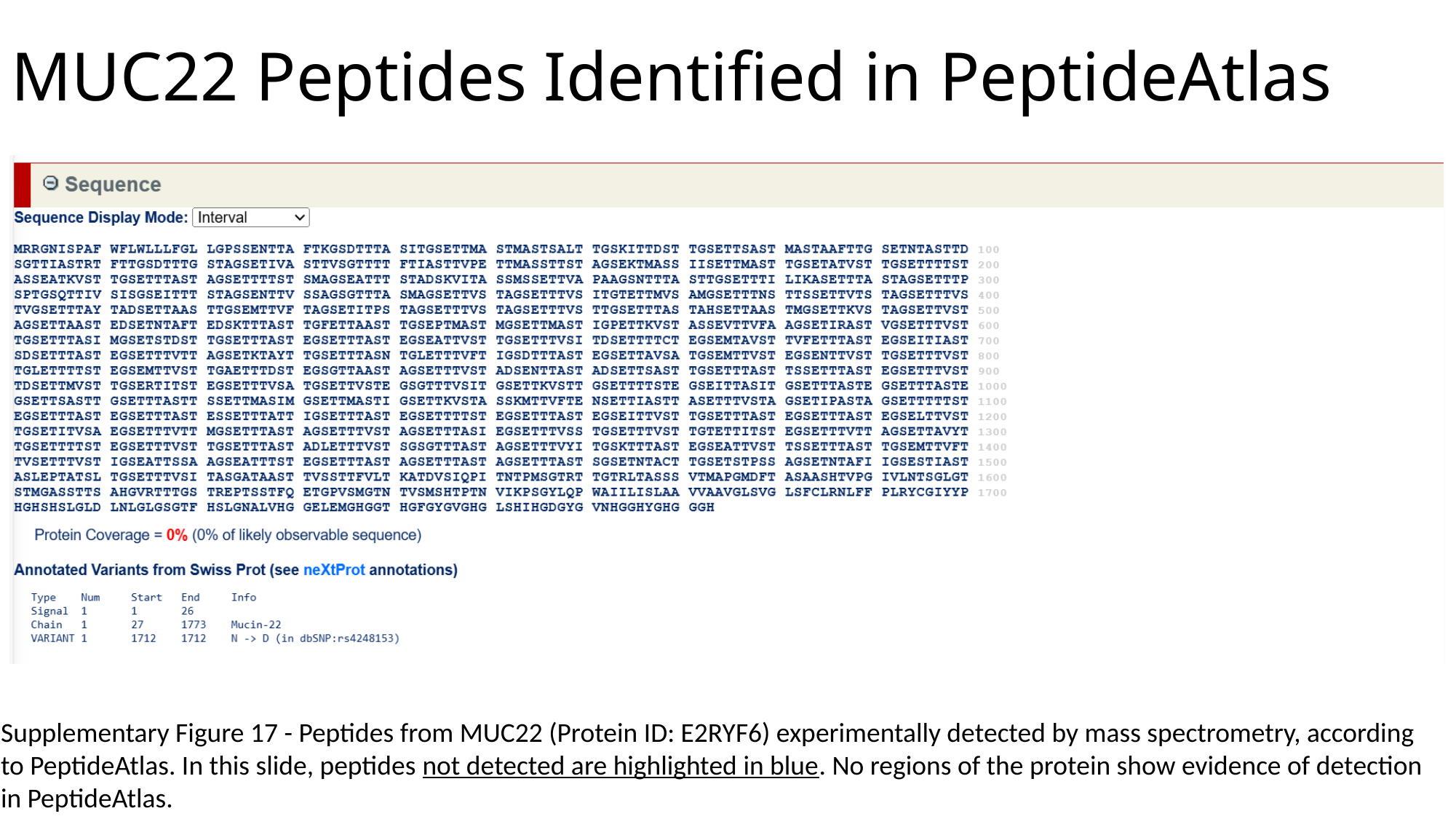

# MUC22 Peptides Identified in PeptideAtlas
Supplementary Figure 17 - Peptides from MUC22 (Protein ID: E2RYF6) experimentally detected by mass spectrometry, according to PeptideAtlas. In this slide, peptides not detected are highlighted in blue. No regions of the protein show evidence of detection in PeptideAtlas.
